# Techno-Economic and Environmental Assessment of Solar-Driven Hybrid Adsorption Desalination–HDH Using Silica Gel/Cacl_2_ Under Saudi Arabian Climate

**DOI:** 10.3390/gels12030226

**Published:** 2026-03-10

**Authors:** Ehab S. Ali, Ahmed S. Alsaman, Ridha Ben Mansour, Rached Ben-Mansour

**Affiliations:** 1 Interdisciplinary Research Center for Sustainable Energy Systems, King Fahd University of Petroleum and Minerals, Dhahran 31261, Saudi Arabia; ehab.abdelaal@kfupm.edu.sa (E.S.A.); ridha.benmansour@kfupm.edu.sa (R.B.M.); rmansour@kfupm.edu.sa (R.B.-M.); 2 Mechanical Engineering Department, Faculty of Engineering, King Fahd University of Petroleum and Minerals, Dhahran 31261, Saudi Arabia

**Keywords:** adsorption desalination, silica gel/CaCl_2_ composite, dried-gel adsorbents, ejector, HDH, heat recovery, techno-economic analysis, environmental assessment, Saudi Arabia

## Abstract

This study explores a solar-driven hybrid desalination approach developed for Saudi Arabian climatic conditions, combining adsorption desalination (AD) based on a silica gel/CaCl_2_ composite with an ejector (EJ) and a HDH system. The proposed integration aims to enhance vapor utilization and reuse condenser heat to generate additional distillate. Two operating modes are examined, including a productivity-focused strategy that activates evaporator/condenser heat recovery (HR) when cooling is not required. Compared to raw silica gel (SG), the composite adsorbent improves adsorption cycle performance, raising the COP from about 0.38–0.43 to 0.55–0.63, and increasing SCP from roughly 130–240 W/kg to 320–675 W/kg. Without HR, the full AD–EJ–HDH system achieves SDWP of 52–100 m^3^/ton·day with GOR of 2.40–2.75 over the year. In HR-enabled operation, SDWP increases to 81–140 m^3^/ton·day and GOR rises to 2.7–2.95, reflecting stronger internal heat reuse and improved vapor management. Techno-economic results show that the solar-driven unit cost for AD–EJ–HDH decreases from winter values (2.7–2.9 $/m^3^) to a minimum around June (1.53 $/m^3^), while waste heat operation reduces the cost further to 0.49 $/m^3^ in June (rising to ~0.76–0.80 $/m^3^ in winter). With HR, the full AD–HR–EJ–HDH reaches around 1.44 $/m^3^ (solar, June) and 0.38–0.40 $/m^3^ (waste heat, summer), confirming the advantage of desalination-focused HR operation when cooling is not required. Finally, compared with SWRO, the AD–HR–EJ–HDH configuration delivers an approximately 90% lower carbon footprint on the same environmental assessment basis. The study highlights the environmental benefit of the intensified SG/CaCl_2_ hybrid configuration.

## 1. Introduction

Freshwater scarcity is intensifying across arid and semi-arid regions as population growth, industrial expansion, and climate warming increase demand while constraining renewable supplies [1,2,3]. In Saudi Arabia, the imbalance is structural: natural freshwater availability is limited, and the national water system depends heavily on seawater desalination to meet municipal and industrial needs. Current national production capacity is reported to be 11.5 million m^3^/day, highlighting both the strategic importance of desalination and the scale at which energy and environmental impacts accumulate [4].

At the global level, desalination is dominated by seawater reverse osmosis (SWRO) and thermal technologies such as multi-effect distillation (MED) and multi-stage flash (MSF). SWRO has steadily improved through membranes and energy recovery devices, and recent reporting places typical SWRO specific energy consumption (SEC) around 2.5–4.0 kWh/m^3^, with a thermodynamic minimum that is much lower [5]. Nevertheless, electricity-driven desalination remains tightly coupled to grid carbon intensity; this coupling is particularly relevant in Saudi Arabia, where the grid emission factor used in climate-accounting frameworks has been reported as 0.568 tCO_2_/MWh (combined margin) [6].

Beyond greenhouse gases, desalination can affect marine receiving waters through elevated salinity and temperature and through residual treatment chemicals in the discharge stream. The concern is acute for the Arabian/Persian Gulf, a semi-enclosed basin surrounded by desalination infrastructure; modeling and synthesis studies report measurable local salinity/temperature perturbations near the southern Gulf coastline and emphasize cumulative risks as capacity expands [7,8].

These realities have accelerated interest in renewable-driven and low-carbon desalination routes. Saudi Arabia is well-positioned for solar utilization: long-term analyses of surface solar radiation over Saudi Arabia (2013–2021) indicate high annual mean values (e.g., 459 W/m^2^ all-sky, with substantially higher clear-sky means reported) [9]. However, deployment is not only a question of resources. In hot-arid environments, dust and soiling can cause sizeable performance losses for solar collectors and photovoltaic surfaces, with the magnitude depending strongly on on-site conditions, cleaning strategy, and particle loading [10]. Consequently, solar desalination concepts that (i) can use low-grade heat, (ii) remain efficient at moderate driving temperatures, and (iii) can be hybridized to recover internal heat are increasingly attractive for robust operation under Gulf climates [11,12,13,14].

Among emerging thermally driven options, adsorption desalination (AD) has drawn attention because it can convert low-temperature thermal energy [15,16] (solar thermal, industrial waste heat, engine jacket heat) into freshwater production, often with the co-benefit of chilled water generation [17,18,19]. The AD cycle relies on cyclic adsorption–desorption of water vapor on a porous solid sorbent, coupled with an evaporator (seawater feed) and condenser (freshwater collection) [20,21,22,23]. Its theoretical appeal is strengthened by practical advantages: operation at relatively low pressures, the absence of high-salinity boiling brine inside heat transfer surfaces (mitigating scaling compared to high-temperature thermal plants), and the potential to valorize waste heat while producing two “products” (water and cooling) [24,25,26,27]. In many designs, the key barrier has not been feasibility but rather specific productivity and thermal efficiency, which are controlled by sorbent working capacity, mass/heat transfer in the bed, and the degree of internal heat recovery [28,29]. A number of studies have proposed using fluidized-bed configurations to enhance the performance of the AD system. For instance, Krzywanski et al. [30] introduced a fluidized-bed strategy to address the intrinsic heat and mass transfer restrictions of conventional adsorbent beds, reporting improved transport characteristics through optimization of bed hydrodynamics and thermal behavior. In a related investigation [31], the effects of key operating and material parameters, particularly adsorbent particle size and the velocity of the fluidizing air, were systematically examined. Lasek et al. [32] also presented a review of fluidized-bed applications, emphasizing their strong potential to markedly improve overall system efficiency. Additionally, Krzywanski et al. [33] employed machine learning to identify the optimal fluidization conditions for the AD system.

Silica gel (SG) is a prototypical dried gel (xerogel) with a nanoporous network and surface silanol chemistry that yields strong affinity for water vapor. Its adsorption characteristics may be controlled through control of the pore size distribution, surface functional groups, and thermal conductivity modifiers. Recent studies on the subject of materials have shown the following results on the translation of microstructure to desalination performance: nanoporous silica materials have shown SDWP of 25 kg/kg-adsorbent·day and GOR of 0.77 (in the context of the adsorption cooling/desalination cycle), indicating the sensitivity of the performance to the sorbent rather than the arrangement of the cycles [34].

Besides the aforementioned “pure” silica, the most popular way to increase the water adsorption capacity of the adsorbent material at low regeneration temperatures is to add hygroscopic salts to the silica matrix to produce a composite gel adsorbent material. The problem of using the silica gel/CaCl2 composite adsorbent is the stability and heat transfer characteristics of the composite material, which may experience salt agglomeration, deliquescence, and the resulting heat transfer characteristics. To overcome the aforementioned difficulties, composite materials have been developed that have a higher thermal conductivity and an adsorption capacity that increases “multiple fold” relative to the silica gel [35].

Even though there have been consistent improvements reported on the adsorbents and the structure of the adsorption beds, traditional silica gel-based AD systems have frequently reported low freshwater productivity on a per mass basis of adsorbent material used. For instance, there have been reported performance syntheses and experimental evaluations of AD systems with SDWP values around 10 m^3^/ton·day, which have been improved through heat/mass recovery approaches. An experimental study reported that the AD system achieved 9.58 m^3^/ton·day for the modified system, which is around 66% more than the conventional two-bed AD system [36].

Earlier system-level developments also include condenser–evaporator heat recovery concepts that were reported to raise silica gel AD freshwater productivity, but such systems are not universal and depend on driving temperature, cycle time, and bed heat transfer [37].

To overcome the remaining productivity and efficiency constraints, recent research has increasingly focused on hybridization, integrating AD with complementary thermal cycles that either (i) upgrade available heat, (ii) enhance vapor generation/entrainment, or (iii) recover sensible/latent heat that is otherwise rejected. Two hybrid routes are especially relevant for solar-driven operation in hot climates:

First, ejectors provide compression/entrainment without moving parts and can intensify vapor transport when designed around low-grade heat sources. Many AD-ejector configurations have been studied to enhance freshwater production and gain the output ratio [38]. A representative adsorption–ejector desalination (ADEJ) study reported that applying internal heat recovery and operating at 95 °C desorption temperature could deliver SDWP around 52.67 m^3^/ton·day, about 5 times that of a standalone adsorption desalination system, while also increasing COP (reported COP about 1.47 in the same work) [39]. These results motivate ejector-assisted AD architectures as a pathway to raise productivity without relying on high electrical input, an important advantage when the objective is carbon reduction.

Second, HDH is coupled with AD, where multiple forms of synergy emerge: cooled streams from the AD evaporator can enhance dehumidification, while recovered heat from adsorption components can elevate humidifier inlet conditions, raising water production per unit driving heat [40,41,42,43].

Recent integrated studies show the extent of these benefits of hybridization, especially when using silica gel/CaCl_2_ for lifting working capacity. A solar-powered study on AD–HDH using silica gel/CaCl_2_ found that the best combined configuration could attain SDWP up to 69 m^3^/ton·day during June, along with GOR up to 1.8 for a specific integrated configuration, with substantial reductions in water cost when waste heat is employed [44]. The cost values for freshwater cost could fall as low as approximately 2.03 $/m^3^ and as low as 0.53 $/m^3^ when waste heat is employed for an optimized combined configuration. These benefits are not only energy-related but also encompass the combined benefits of higher capacity/kinetics of the sorbent as well as enhanced internal heat recovery of sensible as well as latent heat, thus showing a strong link towards the gel scope for “materials-to-systems” for the combined benefits of dried gel materials for energy as well as environmental technologies. A further step is tri-hybridization (AD + ejectors + HDH), which seeks to compound the above synergies. In one tri-hybrid report, the integrated cycle with heat recovery delivered SDWP 83.1 m^3^/ton·day with an overall GOR of about 2.75 and a reported freshwater specific cost of 1.49 $/m^3^ [45]. These figures position hybrid sorption-based desalination among the more competitive solar thermal-compatible pathways in terms of productivity per unit sorbent mass, while preserving operation at moderate driving temperatures.

Despite these strong advances, three gaps remain evident:

1. Materials–systems gap for gel-based composites. While silica gel/CaCl_2_ has been positioned as a high-capacity composite for AD–HDH hybrids (explicitly framed as “first time” for AD–HDH in that study), the tri-hybrid AD–EG–HDH literature has largely been developed around silica gel rather than silica gel/CaCl_2_ composites. This leaves an open question: how a composite dried gel adsorbent (silica gel/CaCl_2_) translates into system-level gains when ejector intensification and HDH waste heat reuse are applied simultaneously.
2. Climate-specific design gap for Saudi Arabia. The most closely related hybrid demonstrations and assessments in the provided literature are conducted under Assiut, Egypt, weather conditions. However, Saudi Arabian operation (ambient temperature profile, humidity patterns along the Red Sea and Arabian Gulf coasts, and solar resource characteristics) can shift the optimal cycle timing, heat recovery benefits, and collector/storage sizing. A Saudi climate-based evaluation is therefore required to establish realistic annual performance and economics.
3. Incomplete sustainability reporting in hybrid studies. Many hybrid studies emphasize thermal metrics and water cost (e.g., SDWP, GOR, and $/m^3^), but environmental indicators are less consistently integrated into the same framework—particularly those that are location-dependent (e.g., CO_2_ intensity linked to the local electricity mix and auxiliary power demand). This is a nontrivial omission for deployment in KSA, where desalination sustainability is increasingly judged by both cost and decarbonization potential.

Taken together, the literature supports the technical promise of (i) composite gel adsorbents in AD–HDH and (ii) ejector intensification in adsorption-based desalination, yet it still lacks a unified AD–EG–HDH assessment built on silica gel/CaCl_2_, evaluated under the Saudi Arabian climate, and accompanied by a techno-economic and environmental appraisal.

### Aim and Contributions of the Present Study

This study presents a techno-economic and environmental assessment of a solar-driven hybrid AD–EG–HDH desalination system employing a silica gel/CaCl_2_ composite adsorbent under Saudi Arabian climate conditions.

The main contributions are:

− The present work investigates, to the best of the authors’ knowledge, for the first time, the combined AD–EG–HDH configuration using silica gel/CaCl_2_ (i.e., merging composite gel enhancement with ejector intensification and HDH).
− The system is evaluated under representative Saudi meteorological conditions to quantify seasonal/annual freshwater productivity and heat utilization, moving beyond the closely related assessments conducted under Assiut, Egypt, weather inputs.
− The work converts performance outcomes into cost metrics (e.g., water unit cost and sensitivity to key design/operational parameters), in a manner comparable to prior hybrid cost reporting (e.g., ~2 $/m^3^ for AD–HDH with silica gel/CaCl_2_ in peak months and 1.49 $/m^3^ for tri-hybrid silica gel AD–EG–HDH with heat recovery), while reflecting the Saudi operating context.
− Environmental assessment aligned with deployment needs. Alongside thermal/economic metrics, the study quantifies environmental indicators (e.g., CO_2_ implications linked to auxiliary electrical loads and operating strategy), enabling a consistent comparison of “performance–cost–environment” tradeoffs for KSA deployment.

## 2. Results and Discussion

The Results and Discussion Section assesses the viability of the suggested solar-driven hybrid AD-EJ-HDH system, which employs the silica gel/CaCl2 composite adsorbent, under the climate conditions of Saudi Arabia, with special reference to seasonal variations in the climate. It initially presents the results in the form of adsorption cycle performance (COP and SCP), and then presents the results in the form of system-level performance (SDWP and GOR), including the HR mode of operation when the system is not required to operate in the cooling mode.

**Figure 1:**
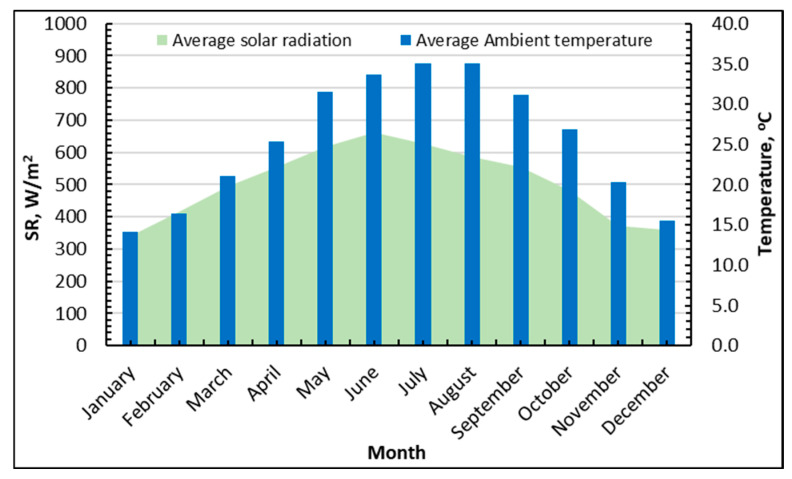
Average monthly solar radiation and ambient temperature extracted from TRNSYS software (version 18.0) for Riyadh, Saudi Arabia.

Figure 1 presents the monthly climate boundary conditions employed to assess the solar-driven hybrid system’s viability. SR increases with the seasons from winter to early summer, reaching a maximum of 670 W/m^2^ in June from 340 W/m^2^ in February and January. Thereafter, SR remains relatively high through late summer (≈580–620 W/m^2^ in July–August) before declining toward 360 W/m^2^ in December. In parallel, the ambient temperature increases from 14–16 °C in January–February to 30–33 °C in May–June, reaching its highest level in mid-summer at 35 °C in July–August, and then decreases again to winter values by the end of the year. Overall, the figure indicates that the climate in Riyadh is characterized by a long hot season with simultaneously strong solar availability—conditions that are favorable for solar thermal driving but challenging for heat rejection.

**Figure 2:**
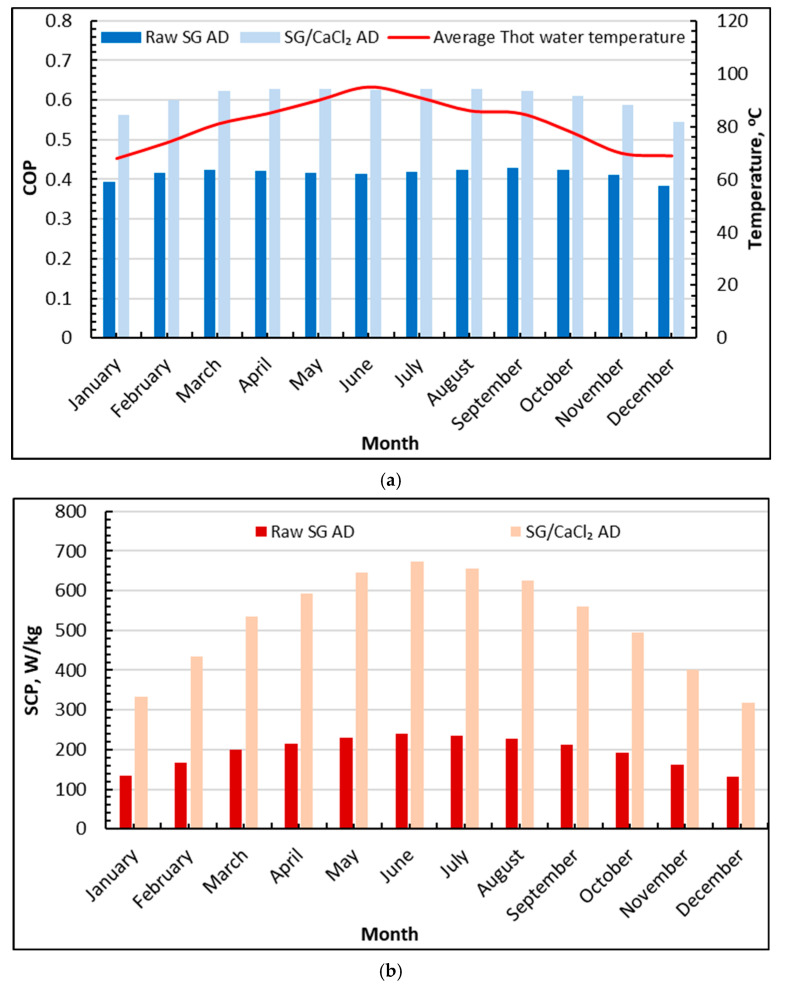
Average monthly SCP and COP of silica gel-CaCl_2_ AD compared to raw silica gel: (**a**) COP; (**b**) SCP.

Figure 2a shows that the average hot water temperature increases from winter to early summer, rising from about 68 °C in January to a peak of 95 °C in June, then declining to 69 °C by December. Over the same period, the COP of the adsorption desalination (AD) cycle remains consistently higher when silica gel/CaCl_2_ is used. For the case involving raw silica gel, the values stay within the range of 0.38 to 0.43. The lowest value occurs in December and reaches 0.38. The maximum value occurs in August and September and reaches 0.43. For the case involving silica gel and CaCl_2_, the values range between 0.55 and 0.63. The values start at 0.56 in January and reach their maximum at 0.63 in June. The values gradually decrease to 0.55 in December.

The seasonal pattern shows that higher hot water temperatures driven by the sun increase the rate of desorption and thus increase the effective adsorption–desorption swing to improve the useful effect per unit heat supplied to the adsorbents. The limited range of the COP values for the case involving raw silica gel shows that the useful effect is limited by the working capacity and transport resistances in the bed level. Therefore, increasing the hot water temperature improves the COP to a limited extent. However, the case involving silica gel and CaCl_2_ shows visibly higher COP values throughout the year. The values are 40% higher than the values for the case involving raw silica gel.

Figure 2b presents the monthly average SCP (W/kg), which reflects the cycle’s cooling-side intensity and, indirectly, the rate of vapor generation/evaporation that supports desalination. For raw silica gel, SCP increases from about 135 W/kg in January to a peak near 240 W/kg in June, then declines again toward 130 W/kg in December. The silica gel/CaCl_2_ composite exhibits a much larger magnitude and stronger seasonal response, rising from about 330 W/kg in January to approximately 675 W/kg in June, followed by a gradual decrease to about 320 W/kg in December. The composite delivers an SCP that is about 3.5 times that of raw silica gel in June.

This marked enhancement is consistent with the role of CaCl_2_ impregnation in increasing the sorbent’s effective water uptake and sustaining higher adsorption rates under the same boundary conditions. In practical terms, higher SCP indicates that the evaporator can absorb heat more intensively (i.e., higher evaporation/latent heat transfer rate), enabling a more vigorous cycle and supporting higher freshwater throughput when the evaporated vapor is ultimately condensed.

**Figure 3:**
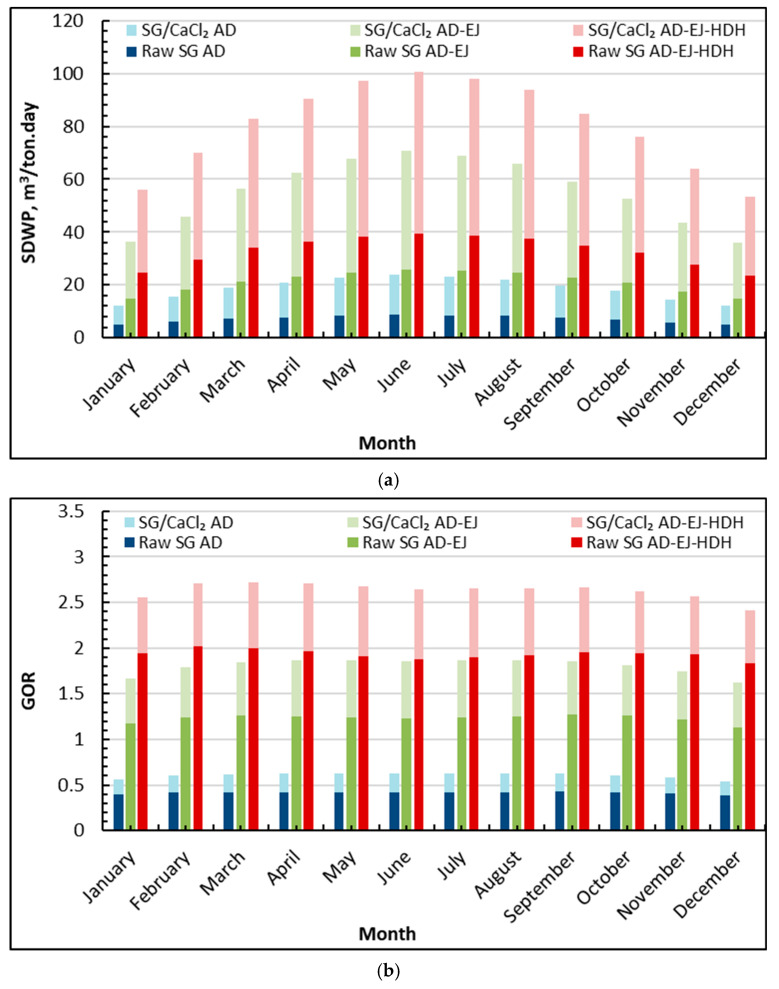
Average monthly SDWP and GOR of silica gel-CaCl_2_ AD-EJ-HDH compared to raw silica gel: (**a**) SDWP; (**b**) GOR.

Figure 3a highlights a strong seasonal response in specific daily water production (SDWP) and a clear performance hierarchy across configurations. For the standalone AD cycle, SDWP remains relatively low, increasing from about 5 m^3^/ton·day (raw SG) and ≈12.3 m^3^/ton·day (SG/CaCl_2_) in January to roughly 8.6 m^3^/ton·day (raw SG) and 23 m^3^/ton·day (SG/CaCl_2_) in June–July. Adding ejector assistance (AD–EJ) raises SDWP markedly: the raw SG AD–EJ case climbs from 15 m^3^/ton·day (January) to ≈25 m^3^/ton·day (June–July), while SG/CaCl_2_ AD–EJ increases from about 36 to 71 m^3^/ton·day over the same period. The highest productivity is consistently achieved by the tri-hybrid AD–EJ–HDH arrangement, where SG/CaCl_2_ AD–EJ–HDH rises from about 55 m^3^/ton·day (January) to a peak close to ≈100 m^3^/ton·day (June–July), then decreases toward 52 m^3^/ton·day (December). In comparison, raw SG AD–EJ–HDH ranges approximately from 25 in winter to 41 m^3^/ton·day at mid-year.

The data also quantify how each enhancement contributes to the overall gain. Relative to standalone AD, the introduction of ejectors increases SDWP by roughly three times for both adsorbents at peak months (e.g., raw SG: ≈11 → ≈34 m^3^/ton·day; SG/CaCl_2_: from 25 to 71 m^3^/ton·day in June), indicating that vapor entrainment and intensified evaporation/condensation substantially elevate the water production rate. Adding HDH on top of AD–EJ delivers a further uplift, most pronounced for the composite adsorbent: at peak season, SG/CaCl_2_ AD–EJ–HDH is about 40% higher than SG/CaCl_2_ AD–EJ, consistent with effective reuse of the adsorption condenser heat for additional water production in the HDH loop. Across all months, the composite sorbent provides a stable advantage over raw silica gel at the same configuration level, about 250% enhancement in SDWP, reflecting the higher working uptake and cycle intensity achievable with silica gel/CaCl_2_, which becomes even more impactful when combined with ejector intensification and HDH heat reuse.

Figure 3b presents the corresponding gained output ratio (GOR), showing that thermal utilization efficiency improves stepwise from AD to AD–EJ, then AD–EJ–HDH, with consistently higher values for SG/CaCl_2_. For standalone AD, GOR remains below unity throughout the year: raw SG AD is approximately 0.38–0.45, while SG/CaCl_2_ AD is higher at about 0.55–0.65. With ejector integration (AD–EJ), GOR increases to around ≈1.20–1.35 for raw SG and about 1.65–1.90 for SG/CaCl_2_, indicating a substantial improvement in the conversion of driving heat into distillate-equivalent output. The highest efficiency is obtained in the AD–EJ–HDH configuration, where raw SG AD–EJ–HDH is roughly 1.85–2.00 across most months, and SG/CaCl_2_ AD–EJ–HDH maintains about 2.40–2.75, with its upper range appearing in late winter–spring (around 2.7) and a slightly lower plateau during the hottest months of about 2.65.

A key insight from Figure 3b is that GOR exhibits milder seasonality than SDWP, implying that the hybrid architecture primarily stabilizes thermal utilization through internal recovery and integration, even as absolute production varies with solar-driven operating conditions. The efficiency advantage of AD–EJ–HDH stems from two complementary mechanisms: (i) ejectors intensify vapor handling and raise the effective distillate output for a given heat input, and (ii) HDH converts a portion of condenser heat, otherwise rejected, into additional freshwater, thereby increasing the “useful output” per unit supplied thermal energy. The composite adsorbent enhances this effect because its higher uptake sustains stronger adsorption–desorption swings at the same boundary temperatures, allowing the system to capitalize more effectively on recovered heat.

**Figure 4:**
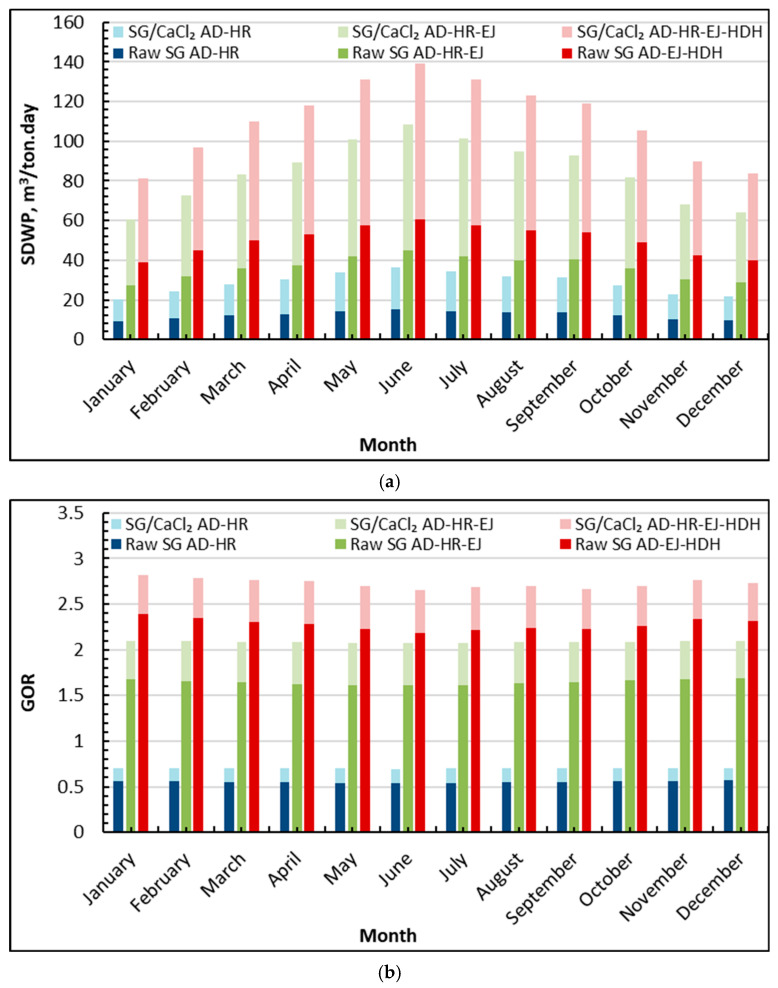
Average monthly SDWP and GOR of silica gel-CaCl_2_ AD-HR-EJ-HDH compared to raw silica gel: (**a**) SDWP; (**b**) GOR.

Figure 4a shows that introducing HR shifts the system toward a productivity-oriented operating mode, with SDWP increasing steadily from winter to early summer and then decreasing toward winter. For the standalone AD-HR, SDWP remains comparatively modest: raw silica gel (SG) increases from about 9 m^3^/ton·day (January) to about 15 m^3^/ton·day (June), while SG/CaCl_2_ rises from roughly 20 to 37 m^3^/ton·day over the same period. When ejectors are added (AD-HR-EJ), the improvement becomes much more pronounced: the raw SG case grows from about 27 m^3^/ton·day (January) to 44 m^3^/ton·day (June), whereas SG/CaCl_2_ reaches approximately 60 in winter and peaks near 109 m^3^/ton·day in June. The highest productivity is consistently obtained with the tri-hybrid AD-HR-EJ-HDH arrangement: SG/CaCl_2_ AD-HR-EJ-HDH increases from about 81 m^3^/ton·day (January) to a peak of 140 m^3^/ton·day (June), then declines to roughly 84 m^3^/ton·day (December). In comparison, raw SG AD-HR-EJ-HDH remains lower, rising from 39–61 in winter to 61 m^3^/ton·day in June.

The trends in Figure 4a are consistent with the role of HR in the adsorption loop: linking the AD evaporator and condenser reduces the pressure ratio Peva/Pcond, which strengthens the cycle’s effectiveness in the amount of vapor that can be adsorbed/desorbed per unit time. This is precisely why HR is adopted when cooling is not required—the operating target becomes maximum distillate production rather than chilled water generation. The additional step changes seen when moving from AD-HR → AD-HR-EJ → AD-HR-EJ-HDH indicate that HR creates a stronger platform for intensification: ejectors amplify vapor handling and entrainment, while the HDH unit converts a portion of the recovered/available condenser heat into additional distillate. Finally, the consistently higher SDWP for SG/CaCl_2_ (often about two times the raw SG value at the same configuration level) reflects how the composite adsorbent’s higher working capacity allows the system to exploit the HR-induced pressure/thermal coupling more effectively—especially during the high-driving months (May–July).

Figure 4b confirms that HR-based operation improves thermal utilization and that efficiency increases stepwise with hybridization. For AD-HR, GOR stays below unity but remains relatively stable: raw SG AD-HR is approximately 0.55–0.65, while SG/CaCl_2_ AD-HR is slightly higher, about 0.65–0.75 across the year. Adding ejectors (AD-HR-EJ) lifts GOR into the range typical of more thermally integrated desalination systems: raw SG AD-HR-EJ is roughly 1.55–1.75, whereas SG/CaCl_2_ AD-HR-EJ reaches about 2.00–2.15 with only mild month-to-month variation. The maximum values occur for AD-HR-EJ-HDH, where raw SG typically lies around 2.18–2.40, while SG/CaCl_2_ AD-HR-EJ-HDH remains close to 2.65–2.81, with the upper end generally appearing outside the hottest months and a slightly lower plateau in peak summer.

The relatively modest seasonality in GOR, compared with SDWP, indicates that HR primarily strengthens how effectively the supplied heat is converted into useful distillate-equivalent output, even as absolute production follows the solar-driven temperature availability. Mechanistically, reducing Peva/Pcond via HR promotes a more favorable internal vapor adsorbed/desorbed; this makes the cycle more thermally coherent and raises the gained output for a given regeneration heat input. The additional increase from EJ and HDH is then logically cumulative: ejectors intensify vapor transport and condensation yield, while HDH upgrades condenser-side heat into extra freshwater rather than rejecting it. The composite adsorbent sustains the highest GOR because its larger uptake and stronger working swing enable more vapor to be processed per cycle at the same driving conditions, meaning that each unit of supplied thermal energy produces more net distillate. The slight efficiency softening during the hottest months is also expected in hot-arid climates: elevated ambient and cooling water temperatures penalize condensation and reduce heat exchanger driving forces, which marginally limit the benefit of recovery-based integration even in HR-focused operation.

**Figure 5:**
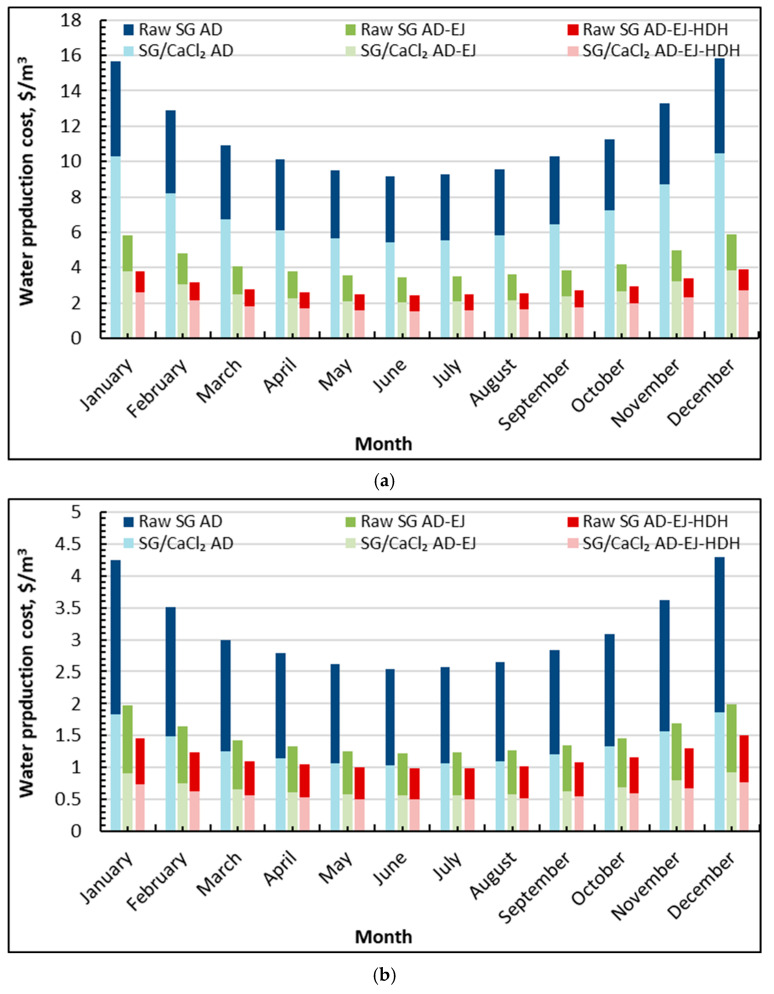
Average monthly freshwater cost and GOR of silica gel-CaCl_2_ AD-EJ-HDH compared to raw silica gel: (**a**) solar powered; (**b**) waste heat.

Figure 5a shows a seasonal pattern in water production cost, with the highest costs in winter and the lowest costs in late spring–summer, consistent with the solar resource and productivity trends. For the baseline raw SG–AD, the cost is the highest among all cases, decreasing from about 15.6 $/m^3^ (January) to a minimum near 9.1 $/m^3^ (June), then rising again to 15.8$/m^3^ (December). Using SG/CaCl_2_ in the same AD configuration reduces the cost substantially, from about 10.3 $/m^3^ (January) to 5.4 $/m^3^ (June), and then back to about 10.5 $/m^3^ (December). When ejectors are integrated (AD–EJ), costs fall further: raw SG AD–EJ drops to roughly 3.4–3.6 $/m^3^ (June–July) (5.8–6.0 $/m^3^ in January/December), while SG/CaCl_2_ AD–EJ reaches approximately 2.03 $/m^3^ (June) (3.7–3.9 $/m^3^ in January/December). The lowest costs are obtained when the HDH stage is added (AD–EJ–HDH): raw SG AD–EJ–HDH is about 2.4 $/m^3^ in June and 3.9 $/m^3^ in winter, whereas SG/CaCl_2_ AD–EJ–HDH reaches the best values at about 1.53 $/m^3^ (June) and remains around 2.7 $/m^3^ in January/December.

From an economic interpretation perspective, the ordering in Figure 5a closely mirrors the productivity hierarchy observed earlier (AD < AD–EJ < AD–EJ–HDH and SG/CaCl_2_ outperforming raw SG). The solar-powered case carries the additional burden of solar field and storage costs; therefore, months with higher solar availability and higher SDWP dilute the capital-related cost per unit water, producing the summer minima. The composite adsorbent and hybridization deliver a compounding benefit: at mid-year, shifting from raw SG–AD (9.1 $/m^3^) to SG/CaCl_2_ AD–EJ–HDH (1.53 $/m^3^) corresponds to an approximate 85% reduction in water cost, while even relative to SG/CaCl_2_–AD (5.4$/m^3^), the tri-hybrid option reduces cost by roughly 75%.

Figure 5b presents the same configurations when the system is driven by waste heat, and the overall cost levels drop markedly across all months. The baseline raw SG–AD decreases from about 4.3 $/m^3^ (January/December) to a minimum near 2.5 $/m^3^ (June). The SG/CaCl_2_–AD case is much lower, ranging approximately from ≈1.85 $/m^3^ (January/December) down to about 1.03 $/m^3^ (June). With ejector integration, costs decline further: raw SG AD–EJ falls to about 1.2 $/m^3^ (June), while SG/CaCl_2_ AD–EJ reaches roughly 0.56 $/m^3^ (June). The best-performing case remains SG/CaCl_2_ AD–EJ–HDH, which achieves the minimum costs of about 0.49 $/m^3^ (June) and increases to about 0.76$/m^3^ in January/December; the corresponding raw SG AD–EJ–HDH is typically around 1.0 $/m^3^ in summer and 1.50 $/m^3^ in winter.

The waste heat results emphasize that the cost sensitivity shifts once the thermal energy is treated as low-cost (or recovered) rather than supplied by a dedicated solar field. Seasonal variation still exists, reflecting month-to-month changes in system productivity and heat rejection conditions, but the amplitude is smaller than in the solar-powered case because the dominant capital term associated with solar collection is reduced or removed. Importantly, the relative value of hybridization becomes even clearer: at peak conditions, moving from raw SG–AD (2.5 $/m^3^) to SG/CaCl_2_ AD–EJ–HDH (about 0.49 $/m^3^) corresponds to an approximate 83% reduction. Even against SG/CaCl_2_–AD (1.0 $/m^3^), the tri-hybrid option cuts the cost by about 55%. In practical deployment terms, Figure 5b indicates that coupling the SG/CaCl_2_ adsorption cycle with ejector intensification and HDH heat reuse can push the system into a low-cost regime below 0.50 $/m^3^ in favorable months when waste heat is available, supporting the argument that the proposed configuration is particularly attractive for industrial/coastal sites where low-grade heat streams can be recovered and converted into freshwater with high overall thermodynamic utilization.

**Figure 6:**
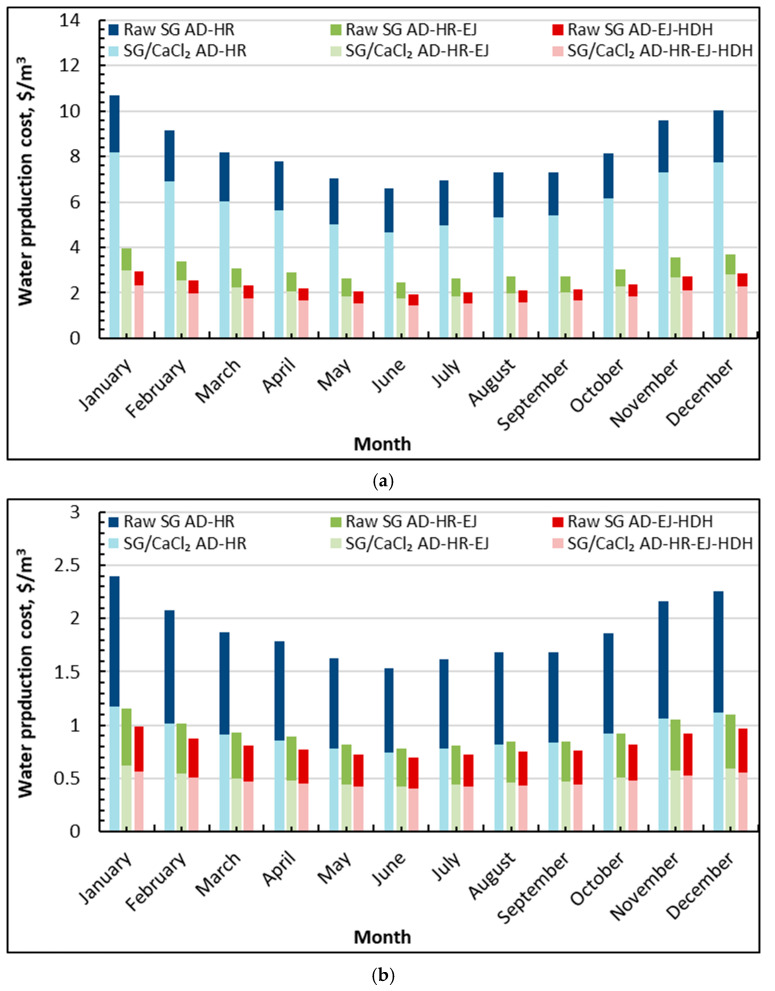
Average monthly freshwater cost and GOR of silica gel-CaCl_2_ AD-HR-EJ-HDH compared to raw silica gel: (**a**) solar powered; (**b**) waste heat.

Figure 6a compares the water production cost for HR-driven operation, where HR links the AD evaporator and condenser to reduce Peva/Pcond and increase the adsorbed/desorbed vapor—applied when a cooling effect is not required. The seasonal trend is clear for all cases: costs decrease from winter toward early summer (higher solar input and higher productivity), then increase again toward winter. The highest costs occur for the HR-only adsorption configurations, especially raw SG AD–HR, which declines from about 10.6 $/m^3^ (January) to 6.6 $/m^3^ (June) and rises again to 10.0 $/m^3^ (December). Using SG/CaCl_2_ in the same HR-only mode reduces the cost noticeably, from roughly 8.2 $/m^3^ (January) to about 4.66 $/m^3^ (June), then back to 7.75 $/m^3^ (December). Once ejectors are included (AD–HR–EJ), the cost drops sharply: raw SG AD–HR–EJ remains around 2.47–3.8 $/m^3^ (minimum near 2.47 $/m^3^ in June), while SG/CaCl_2_ AD–HR–EJ reaches about 1.75 $/m^3^ (June). The lowest costs are obtained when the fully integrated configuration is used (HR + EJ + HDH), where SG/CaCl_2_ AD–HR–EJ–HDH falls to about 1.44 $/m^3^ (June) and remains around 2.2–2.3 $/m^3^ in the coldest months; the corresponding raw-sorbent tri-hybrid case is higher, typically 1.95–2.8 $/m^3^ across the year.

The economics are consistent with the physical role of HR and the hierarchy of intensification. In solar-powered operation, the water cost is strongly influenced by the monthly freshwater output because collector and storage costs are effectively over the produced volume. The HR linkage reduces the internal pressure ratio (lower Peva/Pcond), which increases the vapor exchange per cycle and therefore raises SDWP; this directly lowers the unit water cost compared with non-enhanced adsorption operation. However, the largest cost reductions emerge when ejectors and HDH are added: ejectors intensify vapor handling and condensation yield, while HDH converts part of the rejected condenser heat into additional distillate. Under these conditions, the SG/CaCl_2_ composite provides a compounding advantage because the higher working uptake allows the HR-driven pressure/thermal coupling to be converted into a larger effective throughput, particularly during the high-driving months. Quantitatively, at peak season, the shift from raw SG AD–HR (6.5 $/m^3^) to SG/CaCl_2_ AD–HR–EJ–HDH (1.44 $/m^3^) represents roughly an 80% reduction, illustrating how HR (in desalination-only mode) becomes most valuable when combined with system-level heat and vapor recovery.

Figure 6b presents the same HR-based configurations when the regeneration heat is treated as waste heat, and the absolute costs drop substantially with a weaker seasonal amplitude than the solar-powered case. For raw SG AD–HR, the cost decreases from about 2.4 $/m^3^ (January) to a minimum near 1.5 $/m^3^ (June), then increases to around 2.3 $/m^3^ (December). The SG/CaCl_2_ AD–HR case is consistently lower, reaching approximately 0.74 $/m^3^ (June) and rising to about 1.1–1.2 $/m^3^ in winter. Adding ejectors (AD–HR–EJ) reduces the cost further: raw SG AD–HR–EJ is about 0.78 $/m^3^ (June) and 1.1$/m^3^ in winter, while SG/CaCl_2_ AD–HR–EJ drops to roughly 0.43 $/m^3^ (June) and stays near 0.62 $/m^3^ in winter. The minimum costs are achieved by the fully integrated HR-based tri-hybrid, where SG/CaCl_2_ AD–HR–EJ–HDH reaches about 0.41$/m^3^ (June) and remains around 0.57 $/m^3^ in winter; the corresponding raw-sorbent case is higher, typically around 0.7–1.0 $/m^3^ depending on the month.

The waste heat scenario clarifies the benefit of integration, because the cost is less dominated by the solar field investment and more governed by system productivity and auxiliary requirements. Even when heat is recovered rather than purchased, HR remains advantageous in desalination-only mode because linking the evaporator and condenser reduces Peva/Pcond and supports higher vapor circulation, thereby lowering the cost per cubic meter. Yet the most decisive gains again come from combined intensification: ejectors increase vapor processing capacity, and HDH upgrades condenser heat into additional distillate, pushing costs into the sub-0.5 $/m^3^ range for SG/CaCl_2_ AD–HR–EJ–HDH during favorable months. In other words, Figure 6b shows that when low-grade heat is available, the proposed HR-based integration does not merely reduce cost marginally; it can shift the system into a distinctly low-cost operating regime while preserving the thermodynamic rationale of HR (no cooling requirement, productivity maximization).

Table 1 benchmarks the present AD–EJ–HDH configuration (Riyadh, SG/CaCl_2_) against closely related adsorption–ejector–HDH and adsorption–HR–HDH systems reported in the literature. For the present study, introducing the HR-enabled configuration (AD–HR–EJ–HDH) increases freshwater productivity (SDWP rises from 100 to 140 m^3^/ton·day) and further reduces the unit freshwater cost (from 1.53 and 1.44 $/m^3^ under solar operation for the two configurations with and without evaporator/condenser HR). This highlights the potential of the proposed system compared to the systems reported in the literature.

Table 2 provides a clear quantitative picture of how hybridization progressively reduces the effective electricity demand of the investigated SG/CaCl_2_ configurations (basis: 10,000 m^3^/year over 30 years). The baseline adsorption desalination case (AD) is set at 1.38 kWh/m^3^ for electrical energy consumption, corresponding to 13,800 kWh/year [47]. Introducing the HR mode (evaporator–condenser linking in the AD loop, applied when cooling is not required) lowers the specific consumption to 1.195 kWh/m^3^ (11,947 kWh/year). The most pronounced step change occurs once ejector assistance is added: AD–EJ reduces the specific consumption to 0.465 kWh/m^3^, and further integration yields 0.402 kWh/m^3^ for AD–HR–EJ. When the HDH unit is coupled to valorize rejected heat for additional distillate production, the electricity demand drops to 0.319 kWh/m^3^ for AD–EJ–HDH, reaching the minimum of 0.308 kWh/m^3^ for the fully integrated AD–HR–EJ–HDH configuration. Relative to the baseline AD (1.38 kWh/m^3^), the full hybrid system therefore achieves an electricity reduction of about 78%, confirming that combining HR (desalination-only mode), ejector intensification, and HDH heat reuse substantially improves overall energy effectiveness.

These improvements translate directly into lower lifecycle emissions under a fossil-based electricity supply. Over 30 years, the baseline AD corresponds to 115.9 tCO_2_ (gas) or 132.5 tCO_2_ (oil), whereas the best-performing AD–HR–EJ–HDH case decreases emissions to 25.8 tCO_2_ (gas) and 29.5 tCO_2_ (oil)—again a reduction close to ~78%. Importantly, the hybrid configurations also compare favorably against conventional desalination benchmarks listed in the same table. For instance, SWRO is shown in the range 3.0–4.0 kWh/m^3^, while MED and MSF span 5.5–9.0 and 10.0–16.0 kWh/m^3^, respectively. Against these references, the AD–HR–EJ–HDH configuration at 0.308 kWh/m^3^ is lower by roughly 90% versus SWRO and by an even larger margin versus MED/MSF, emphasizing the potential of solar-driven, thermally integrated sorption-based desalination to decouple freshwater production from high electrical consumption.

The same trend is reflected in the monetized carbon term (“carbon credit/cost equivalent”) reported in Table 2. For gas-based emissions, the 30-year value declines from $4637 (AD) to $1034 (AD–HR–EJ–HDH), while for oil, it drops from $5299 to $1181. In practical terms, this means that moving from a basic adsorption configuration to the fully integrated hybrid reduces not only the electrical burden but also the associated long-term carbon liability (or equivalently increases the avoided-carbon benefit if interpreted as a credit), strengthening the environmental case for the SG/CaCl_2_ AD–HR–EJ–HDH concept under sustainability-driven desalination planning. Compared with SWRO, the proposed AD–HR–EJ–HDH configuration delivers an approximately 90% lower carbon footprint on the same environmental assessment basis.

Coupling adsorption desalination with HDH can improve brine sustainability by decreasing the reject volume and alleviating high-salinity discharge intensity when benchmarked against a conventional RO plant. This reduction supports (i) lower chemical mass discharge to the marine environment, (ii) enhanced compliance with discharge standards, and (iii) reduced reliance on membrane-cleaning chemicals, given the greater role of thermal recovery and brine valorization in the integrated scheme. The overall chemical demand is projected to be around 35–45% lower than that of a standalone RO facility of the same production capacity” [48].

**Table 2:** Environmental indicators for conventional desalination and investigated SG/CaCl_2_ configurations (10,000 m^3^/year; 30 years) [47,49,50].

Parameter	SWRO	MED	MSF	AD	AD-HR	AD-EJ	AD-HR-EJ	AD-EJ-HDH	AD-HR-EJ-HDH
SEC (kWh/m^3^)	3.0–4.0	5.5–9.0	10.0–16.0	1.380	1.195	0.465	0.402	0.319	0.308
Annual energy (kWh/yr)	30,000–40,000	55,000–90,000	100,000–160,000	13,800	11,947	4648	4023	3185	3077
CO_2_ (gas), t over 30 yr	252–336	462–756	840–1344	115.9	100.4	39.0	33.8	26.8	25.8
CO_2_ (oil), t over 30 yr	288–384	528–864	960–1536	132.5	114.7	44.6	38.6	30.6	29.5
Carbon credit (gas), $	10,080–13,440	18,480–30,240	33,600–53,760	4637	4014	1562	1352	1070	1034
Carbon credit (oil), $	11,520–15,360	21,120–34,560	38,400–61,440	5299	4588	1785	1545	1223	1181

## 3. Conclusions

This study proposed and assessed a solar-driven hybrid adsorption desalination concept under Saudi Arabian climatic conditions, integrating AD with EJ and HDH, and extending the analysis to a desalination-oriented HR mode (applied when cooling is not required). The results show that coupling ejector-based vapor utilization with HDH heat cascading, together with the higher working capacity of silica gel/CaCl_2_, yields substantial gains in productivity, thermal utilization, cost, and environmental indicators across seasonal operation. This is the first techno-economic and environmental assessment of the hybrid AD–EJ–HDH (AD–EG–HDH) architecture using silica gel/CaCl_2_ under Saudi climatic conditions. Key findings of the study include:

- Relative to raw silica gel, the silica gel/CaCl_2_ composite improves the adsorption cycle COP from 0.38–0.43 to 0.55–0.63 and increases SCP from 130–240 W/kg to 320–675 W/kg, indicating faster cycling potential and stronger low-grade heat utilization.
- The fully integrated AD–EJ–HDH configuration achieves SDWP of 52–100 m^3^/ton·day and GOR of 2.40–2.75 over the year, confirming that ejector vapor handling and HDH heat recovery provide a compounded gain beyond AD-only operation.
- When HR is activated by linking the AD evaporator and condenser to reduce Peva/Pcond, the desalination-oriented AD–HR–EJ–HDH configuration increases performance to SDWP of 81–140 m^3^/ton·day and GOR of 2.7–2.95, with pronounced mid-year peaks under favorable conditions.
- For the solar-driven case (without HR), the unit water cost of AD–EJ–HDH declines from about 2.7–2.9 $/m^3^ in winter to 1.53 $/m^3^ in June; under waste heat, the cost decreases to 0.49 $/m^3^ in June (and 0.76–0.80 $/m^3^ in winter). With HR enabled, the full AD–HR–EJ–HDH system reaches about 1.44 $/m^3^ (solar, June) and 0.40 $/m^3^ (waste heat, summer), demonstrating that HR is particularly attractive for desalination-only operation when low-cost thermal energy is available.
- Environmental advantage: Based on a capacity of 10,000 m^3^/year over 30 years, intensification reduces SEC from 1.380 kWh/m^3^ (AD) to 0.308 kWh/m^3^ (AD–HR–EJ–HDH), and decreases total CO_2_ emissions (gas basis) from 115.9 t to 25.8 t, indicating a strong potential for decarbonizing desalination relative to conventional and baseline AD operation.
- Compared with SWRO, the proposed AD–HR–EJ–HDH configuration delivers an approximately 90% lower carbon footprint on the same environmental assessment basis.

Future work should conduct the proposed AD–HR–EJ–HDH configuration experimentally under Saudi coastal and inland conditions, and extend the model to include transient operation, dust impact, optimized control, and thermal storage sizing, brine management/environmental impacts at the outfall, and full lifecycle assessment (LCA) with locally representative electricity and material supply chains. Also it is recommended to study the deliquescence, which can increase mass transfer resistance and reduce effective working capacity.

## 4. Materials and Methods

Silica gel (SG) was selected as the baseline adsorbent due to its wide availability, non-toxicity, and proven suitability for low-temperature adsorption-driven applications. To enhance surface activity and improve adsorption performance, SG was subjected to acid treatment prior to composite preparation. Acid activation using hydrochloric acid has been widely reported as an effective approach for modifying porous adsorbents and increasing their adsorption capacity and specific surface area [51]. Based on these studies, a concentration of 2 M HCl was adopted as it provides the most favorable improvement in adsorption capacity and BET surface area. Accordingly, 60 cm^3^ of 2 M HCl was added to 10 g of SG, and the suspension was stirred at room temperature for 24 h. The treated material was then filtered, thoroughly washed several times to remove residual acid, and dried at 150 °C for 12 h.

The silica gel/CaCl_2_ composite (SG/CaCl_2_) was subsequently prepared using a direct impregnation method. In this procedure, CaCl_2_ was first dissolved in distilled water and then mixed with 2.1 g of dried SG to achieve a salt loading of 25 wt.%. The resulting mixture was stirred at room temperature for 24 h to promote uniform salt distribution within the porous gel structure. After impregnation, the composite was filtered to remove excess solution and then dried at 150 °C for 12 h under atmospheric pressure to eliminate residual moisture and stabilize the composite. The overall workflow of the acid treatment and impregnation steps and detailed synthesis conditions and characterization procedures are provided in the Supplementary File.

### 4.1. System Description

**Figure 7:**
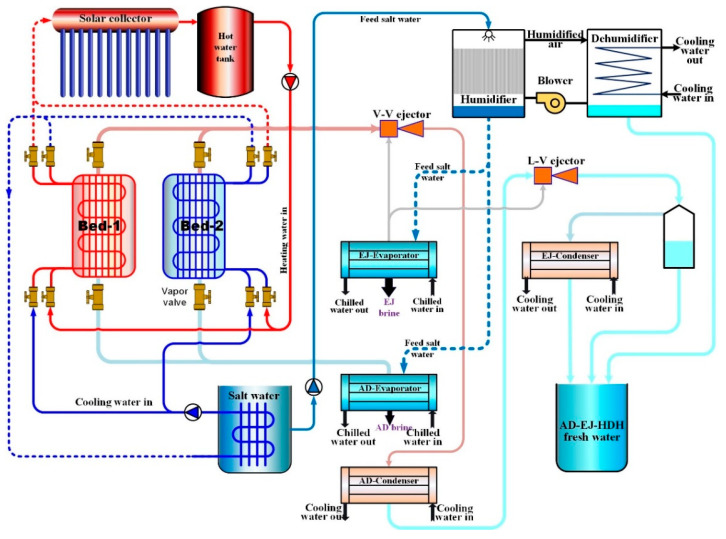
System schematic of SG/CaCl_2_ AD-EJ-HDH hybrid system.

The proposed solar-driven hybrid adsorption desalination–ejectors–HDH (AD–EJ–HDH) system using silica gel/CaCl_2_ as the working adsorbent in a two-bed adsorption cycle is shown in Figure 7. The plant is organized into three tightly coupled subsystems:

1. Solar hot water circuit that supplies the desorption heat to the adsorbent beds.
2. Silica gel with CaCl_2_ composite adsorption desalination unit integrated with two ejectors (vapor–vapor and liquid–vapor).
3. Humidification–dehumidification (HDH) unit that recovers the adsorption condenser’s rejected heat and waste heat resulting from adsorption bed cooling. The HDH could produce additional freshwater production.

The key system-level concept is that the adsorption subsystem produces freshwater directly (via condensation) while simultaneously providing a recoverable heat source (the adsorption condenser) that upgrades the thermal efficiency of the overall plant by driving the HDH loop.

#### 4.1.1. Solar Thermal Supply and Storage

The system is powered by a solar collector loop that heats water and stores it in insulated tanks to enable stable operation beyond instantaneous solar fluctuations. In the reference solar-driven hybrid arrangement, the heated water is stored and then circulated through the beds during desorption, allowing the plant to operate in a quasi-continuous manner across daytime and nighttime periods. Evacuated tube solar collectors have been commonly adopted for this class of systems, with the heated water stored in two tanks to supply the desorption periods of alternating beds. The thermal loop therefore performs two roles: providing the regeneration heat and buffering short-term variability so that the adsorption cycle can be operated with a near-constant driving temperature.

#### 4.1.2. Adsorption Desalination Subsystem (Two-Bed Operation with SG/CaCl_2_)

The adsorption cycle is based on two sorption beds that alternate between adsorption and desorption to sustain continuous vapor uptake and regeneration. A two-bed arrangement is widely used because one bed can adsorb while the other desorbs, ensuring uninterrupted water (and potentially cooling) production. In related adsorption desalination modeling, the beds are treated as heat exchangers packed with sorbent granules.

In the present study, the packed sorbent is silica gel/CaCl_2_ composite, selected because it provides a markedly higher maximum uptake than raw silica gel (e.g., reported W_0_ = 0.95 kg/kg for SG/CaCl_2_ vs. 0.45 kg/kg for raw SG, with improved kinetics parameters in the same source) [44]. This higher working capacity is expected to strengthen the adsorption driving force at a given evaporator/condenser pressure level and, consequently, increase the cycle water throughput when integrated with heat recovery and ejector enhancement.

In the adsorption phase, feed saline water enters the AD evaporator and evaporates at low pressure; the generated vapor is drawn to the bed undergoing adsorption and is captured by the SG/CaCl_2_ sorbent. The evaporator is coupled to a chilled water loop, so evaporation can simultaneously provide a cooling effect when operated at sufficiently low temperatures.

In the desorption phase, the second bed is regenerated by circulating hot water from the solar storage through the bed heat exchanger, desorbing water vapor from the sorbent.

In continuous operation, the hot water and cooling water circuits are switched between beds so that each bed alternates between adsorption (cooling water supplied) and desorption (hot water supplied).

#### 4.1.3. Two-Ejector Enhancement (V–V Ejector and L–V Ejector)

To intensify desalination, the adsorption unit is integrated with two ejectors, whose primary function is to increase vapor generation/entrainment and thereby amplify freshwater productivity relative to a standalone adsorption desalination cycle.A defining feature of the two-ejector architecture is the use of two evaporators:

A low-pressure AD evaporator connected to the sorption beds.

A higher-pressure ejector evaporator (EJ evaporator) that provides the secondary vapor stream to the ejectors.During desorption, the high-pressure vapor regenerated from the hot bed is directed to the V–V ejector as a motive (primary) stream. This motive jet entrains water vapor generated in the EJ evaporator (secondary stream), producing a mixed vapor stream that is routed to the adsorption condenser (AD condenser).

Adsorption condenser as a thermal hub. The mixed vapor from the V–V ejector condenses in the AD condenser, rejecting latent heat that is intentionally recovered to preheat the saline feed entering the HDH unit.

The condensate leaving the AD condenser is then employed as a motive (primary) stream for the L–V ejector, where it mixes with an additional entrained stream coming from the EJ evaporator and exits as a two-phase mixture.

Downstream, the mixture is separated such that the liquid fraction is stored as freshwater, while the vapor fraction is condensed in an ejector condenser and collected as additional product water.

#### 4.1.4. HDH Subsystem and Thermal Coupling with the AD Condenser

The HDH unit consists of a humidifier and a dehumidifier, and its freshwater output corresponds to the reduction in air humidity ratio across the dehumidifier. In this hybrid architecture, the HDH is not driven by a separate heater; instead, it is installed specifically to harvest the adsorption condenser’s waste heat. The saline stream heated by the AD condenser is sprayed in the humidifier, increasing the air temperature and moisture content through direct contact (evaporation into the air stream).

The integration therefore upgrades overall water production by converting condenser heat—otherwise rejected to cooling water—into useful latent/sensible driving potential for the HDH stage.

#### 4.1.5. Hybrid AD-EJ-HDH Operations Modes

**Figure 8:**
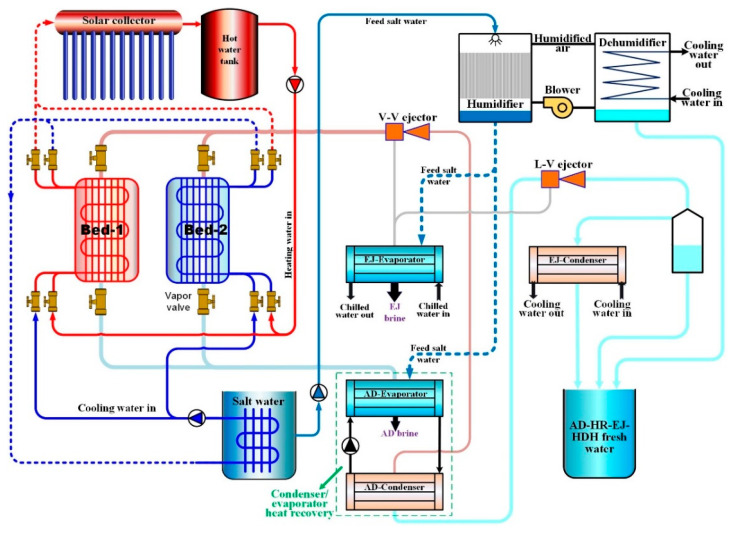
System schematic of SG/CaCl_2_ AD-HR-EJ-HDH hybrid system with evaporator condenser heat recovery (without cooling effect).

Figure 7 and Figure 8 compare two operating configurations that differ by the presence of an evaporator–condenser heat recovery (HR) loop within the adsorption subsystem:

(a) Without evaporator–condenser heat recovery (additional cooling effect).
  In the absence of internal HR, as shown in Figure 7, the AD evaporator can be operated to deliver a useful cooling effect as a by-product, particularly when the evaporator temperature is maintained in the range 7–10 °C. In this mode, the system may simultaneously provide: (i) freshwater from the adsorption/ejector condensers and HDH, and (ii) chilled water output from the AD evaporator, depending on the selected evaporator conditions.
(b) With evaporator–condenser heat recovery (productivity-oriented operation).
  As shown in Figure 8, when cooling is not prioritized, an internal HR circuit can be applied between the AD condenser and AD evaporator, where the latent heat of condensation is reused to enhance evaporation in the AD evaporator. This HR arrangement tends to bring condenser and evaporator pressures closer and increases the adsorption uptake swing, thereby improving freshwater productivity. A similar description is reported for adsorption systems operating with HR, where the evaporator is connected directly to the condenser to exploit the evaporator’s cooling effect for condenser cooling while utilizing condenser heat to intensify evaporation.

While the two-ejector/HDH integration increases vapor generation and recovers rejected heat, the achievable gains ultimately depend on the sorbent’s working capacity and kinetics. In the attached AD–HDH study, switching from raw silica gel to SG/CaCl_2_ is explicitly associated with a “noticeable improvement” in both SDWP and GOR due to the higher adsorption capacity and kinetics of SG/CaCl_2_. Quantitatively, the reported maximum uptake parameter W_0_ nearly doubles (0.45 → 0.95 kg/kg) when CaCl_2_ is incorporated into silica gel.

Accordingly, embedding SG/CaCl_2_ in the beds of the present AD–EJ–HDH system is intended to couple (i) material-level enhancement (larger uptake swing at moderate regeneration temperature) with (ii) system-level intensification (dual-ejector entrainment and condenser-driven HDH). This combination underpins the novelty of investigating the hybrid AD–EJ–HDH layout with silica gel/CaCl_2_ as the working adsorbent.

The silica gel/CaCl_2_ composite characteristics, the governing mass–energy balance model for the AD–EJ–HDH system, and the associated operating assumptions are described in Section 4 (Materials and Methods). Model validation against published benchmark data, along with the full parameter set and supporting calculations, is documented in Section 4 and the Supplementary File for reproducibility.

### 4.2. Governing Equations

The adsorption–ejector (AD–EJ) subsystem considered in this work is modeled in accordance with the framework established in Ref. [45]. Consistent with that approach, a transient formulation is adopted, in which the system dynamics are described through coupled energy balance equations and water vapor adsorption kinetics. The principal governing equations are compiled in Table 3 and Table 4.

The ejector is modeled by applying the conservation of mass, momentum, and energy across its key regions, namely the primary nozzle, suction chamber, mixing section, and diffuser. Within this framework, the governing relations for the flow through the primary nozzle are expressed as follows [45]:

(20) $$P_{p^{\prime }}=P_{p}-\Delta P_{pn}$$

(21) $$h_{p^{\prime },i}=h(P_{p^{\prime }},s_{p})$$

(22) $$h_{p^{\prime }}=h_{p}+\eta_{pn}(h_{p^{\prime },i}-h_{p})$$

(23) $$\nu_{p^{\prime }}=\sqrt{2(h_{p}-h_{p^{\prime }})}$$

Similarly, the equations of the secondary nozzle are written as follows:

(24) $$P_{s^{\prime }}=P_{s}-\Delta P_{sc}$$

(25) $$h_{s^{\prime },i}=h(P_{s^{\prime }},s_{s})$$

(26) $$h_{s^{\prime }}=h_{s}+\eta_{sc}(h_{s^{\prime },i}-h_{s})$$

(27) $$\nu_{s^{\prime }}=\sqrt{2\left(h_{s}-h_{s^{\prime }}\right)}$$

(28) $$P_{s^{\prime }}=P_{p^{\prime }}=P_{m}$$

The velocity and enthalpy of the fluid in the mixing section can be estimated as:

(29) $$\nu_{m}=\eta_{m}\frac{\nu_{s^{\prime }}+ER\cdot \nu_{s^{\prime }}}{1+ER}$$

(30) $$\;h_{m}=\frac{h_{p}+ER\cdot h_{s}}{1+ER}$$

(31) $$s_{m}=s(P_{m},h_{m})$$

The pressure and enthalpy across the normal shock can be given as:

(32) $$P_{as}+\rho_{as}\nu_{as}^{2}=P_{m}+\rho_{m}\nu_{m}^{2}$$

(33) $$h_{as}+\frac{1}{2}\nu_{as}^{2}=h_{m}+\frac{1}{2}\nu_{m}^{2}$$

(34) $$s_{as}=s(P_{as},h_{as})$$

(35) $$h_{e}=h_{m}+0.5\;\eta_{d}\;\nu_{m}^{2}$$

(36) $$P_{e}=P(h_{e},s_{m})$$

This study evaluates an entropy-generation expression to confirm that the model outcomes are thermodynamically admissible and comply with the fundamental constraints imposed by the second law.

(37) $${\dot{S}}_{gen,k}=\sum_{out,k}\left(\dot{m}s\right)-\sum_{in,k}\left(\dot{m}s\right)\geq 0$$

The main relations used in the economic analysis are given in Equations (38)–(42), while the capital cost assumptions for the major components are provided in the Supplementary File.

(38) $${\dot{Z}}_{T}={\dot{Z}}_{c}+{\dot{Z}}_{l}+{\dot{Z}}_{m/o\;}\;\;\;\;\left(in\;\frac{1}{yr}\right)$$

(39) $${\dot{Z}}_{c}=Z_{c}\times F\;\;\;\;\;\;\;\;\left(in\;\frac{\$}{yr}\right)$$

(40) $$F=\frac{r{\left(r+1\right)}^{n}}{{\left(r+1\right)}^{n}-1}\;\;\;\;\;\;\;\;\;\;\;\;\;\;\;\;\;\;\;\;\;\;\;\left(in\;\frac{1}{yr}\right)$$

(41) $${\dot{Z}}_{l}=L\times {\dot{Z}}_{c}\;\;\;\;\;\;\;\left(in\;\frac{\$}{yr}\right)$$

(42) $${\dot{Z}}_{m/o}=M\times {\dot{Z}}_{c}\;\;\;\;\;\;\left(in\;\frac{\$}{yr}\right)$$

The model validation and simulation procedure are attached in the Supplementary File.

## Figures and Tables

**Table 1:** Comparison of desalination performance and freshwater cost for the proposed AD–EJ–HDH configurations versus representative literature systems.

System Configuration	SDWP (m^3^/ton·Day)	GOR	SCP (W/kg)	Freshwater Cost (Solar) ($/m^3^)	Freshwater Cost (Waste Heat) ($/m^3^)
AD–EJ–HDH (without HR) Present study (Riyadh), SG/CaCl_2_	100	2.75	675	1.53	0.49
AD–HR–EJ–HDH Present study (Riyadh), SG/CaCl_2_ [45]	140	2.95	-	1.44	0.40
AD2EJ/HDH + HR (Assiut) silica gel [45]	83.1	2.7	-	1.49	0.54
AD + 2 ejectors + internal HR (AD2EJ-HR), [11], silica gel [45]	67	2.22	-	1.74	0.57
AD + 2 ejectors (AD2EJ) without HR, silica gel, [39,45]	22.9	1.61	220	2.94	1.33
AD-HR, Assiut, Egypt) [44], SG/CaCl_2_	45	0.84	425	3.8	0.63
AD-HR-HDH, Assiut, Egypt), SP/CaCl_2_, [46]	77.3	1.87	-	1.83	0.49
AD–HR-HDH Assiut, Egypt), SG/CaCl_2_, [44]	59	1.8	-	2.0	0.59

**Table 3:** AD SG/CaCl_2_ model [45].

Component	Governing Equation *	Equation
Sorption bed	${\left[{{\left(m\;c_{p}\right)}_{Cu+Al}+\left(m\;c_{p}\right)}_{sp}{+m}_{sg}{c_{p}}_{v}C\right]}^{bed}\frac{{dT}_{bed}}{dt}=m_{SG}H_{st}\frac{dC}{dt}-{\dot{m}}_{w}{c_{p}}_{w}{\left(T_{w,out}-T_{w,in}\right)}^{bed}$	(1)
Heat of adsorption	$H_{st}=h_{fg}+E\left(ln{\left(\frac{C_{o}}{C}\right)}^{\frac{1}{n}}\right)+\frac{E\;T\;\alpha }{n}{\left(\ln\left(\frac{C_{o}}{C}\right)\right)}^{\frac{1-n}{n}}$	(2)
Adsorption evaporator	${\left[{\left(m\;c_{p}\right)}_{Cu}+{\left(mc_{p}\right)}_{w}\right]}^{AD-ev}\;\frac{{dT}_{AD-ev}}{dt}=\theta h_{f}{\dot{m}}_{sw,in}-{\varphi .h}_{fg}{\left(\frac{dC}{dt}\right)}_{ads}m_{sp}+{\dot{m}}_{ch}{c_{p}}_{ch}\left(T_{ch,in}-T_{ch,out}\right)-{\gamma h}_{b}{\dot{m}}_{b}$	(3)
	$m_{sw,ev}\frac{{dX}_{sw,ev}}{dt}={\theta X}_{sw,in}{\dot{m}}_{sw,in}-{\gamma X}_{sw,in}{\dot{m}}_{b}-{\varphi .X}_{D}{\left(\frac{dC}{dt}\right)}_{ads}m_{SG}$	(4)
	$\frac{{dm}_{sw,ev}}{dt}=\theta {\dot{m}}_{sw,in}-\gamma {\dot{m}}_{b}-\varphi {\left(\frac{dC}{dt}\right)}_{ads}m_{SG}$	(5)
Ejector evaporator	${\left\{{\left[{\left(mc_{p}\right)}_{cu}+{\left(mc_{p}\right)}_{w}\right]}^{ev}\;\frac{{dT}_{ev}}{dt}\right\}}_{Ej-ev}={\left\{\theta h_{f}\left(T_{ev},X_{sw,ev}\right)m_{sw,in}^{˙}-h_{fg}\left(ER_{1}+ER_{2}\left(ER_{1}+1\right)\right)\times \varphi {\left(\frac{dC}{dt}\right)}_{des}m_{SG}+{\dot{m}}_{ch}{c_{p}}_{ch}\left(T_{ch,in}-T_{ch,out}\right)-{\gamma h}_{b}\left(T_{ev},X_{s,ev}\right)m_{b}^{˙}\right\}}_{Ej-ev}$	(6)
	${\left[\frac{{dm}_{sw,ev}}{dt}\right]}_{Ej-ev}={\left[\theta \;{\dot{m}}_{sw,in}-\gamma {\dot{m}}_{b}-\left(ER_{1}+ER_{2}\left(ER_{1}+1\right)\right)\times \varphi {\left(\frac{dC}{dt}\right)}_{des}m_{SG}\;\right]}_{Ej-ev}$	(7)
Adsorption condenser	${\left[{\left(m\;c_{p}\right)}_{Cu+ir}+{\left(m\;c_{p}\right)}_{w}\right]}^{AD-co}\;\frac{{dT}_{AD-co}}{dt}=\varphi h_{fg}{\left(\frac{dC}{dt}\right)}_{des}m_{SG}+{\dot{m}}_{m}\;{c_{p}}_{w}{\left(T_{w,in}-T_{w,out}\right)}^{AD-co}$	(8)
Ejector condenser	${\left[{\left(m\;c_{p}\right)}_{Cu+ir}+{\left(m\;c_{p}\right)}_{w}\right]}^{Ej-co}\;\frac{{dT}_{Ej-co}}{dt}=Y.\varphi .h_{fg}\left(1+ER_{1}\right)\left(1+ER_{2}\right){\left(\frac{dC}{dt}\right)}_{des}m_{SG}+{\dot{m}}_{m}\;{c_{p}}_{w}{\left(T_{w,in}-T_{w,out}\right)}^{Ej-co}$	(9)
Outlet temperature from HXs (condensers and evaporators)	$T_{w,out}=T_{hex}+\left(T_{w,in}-T_{hex}\right)\;exp\left(\frac{-{UA}_{hex}}{{\left({\dot{m}c}_{p}\right)}_{w}}\right)$	(10)
Adsorption kinetics	$C_{eq}=C_{o}\exp\left\{-{\left(\frac{RT}{E}\ln\left(\frac{P_{s}}{P}\right)\right)}^{n}\right\}$	(11)
	$\frac{dC}{dt}=\frac{F_{o}D_{s}}{R_{p}^{2}}\left(C_{eq}-C\right)$	(12)
	$D_{s}=D_{so}\;exp\left(\frac{-E_{a}}{RT}\right)$	(13)

* The symbols are defined in the Nomenclature Section.

**Table 4:** The governing equations for the humidifier and dehumidifier [52,53].

Component	Governing Equation *	Equation
Dehumidifier	${{\dot{m}}_{w}}_{HDH}={\dot{m}}_{a}\left(\omega_{in}-\omega_{out}\right)$	(14)
	${\dot{m}}_{a}\left(h_{15}-h_{16}\right)-{{\dot{m}}_{w}}_{HDH}\;h_{13}={\dot{m}}_{sw}\left(h_{18}-h_{17}\right)$	(15)
	$\varepsilon_{deh}=\max\left(\frac{h_{18}-h_{17}}{h_{18,ideal}-h_{17}},\frac{h_{15}-h_{16}}{h_{15}-h_{16,ideal}}\;\right)$	(16)
Humidifier	${{\dot{m}}_{w}}_{HDH}={\dot{m}}_{sw}-{\dot{m}}_{b,HDH}={\dot{m}}_{a}\left(\omega_{out}-\omega_{in}\right)$	(17)
	${\dot{m}}_{a}\left(h_{15}-h_{16}\right)+{\dot{m}}_{b,HDH}\;h_{14}={\dot{m}}_{sw}h_{8}$	(18)
	$\varepsilon_{hum}=\max\left(\frac{h_{15}-h_{16}}{h_{15,ideal}-h_{16}},\frac{h_{8}-h_{14}}{h_{8}-h_{14,ideal}}\;\right)$	(19)

* The symbols are defined in the Nomenclature Section.

## Data Availability

All data generated or analyzed during this study are included in this published article and its Supplementary Information File.

## Supplementary Materials

The following supporting information can be downloaded at: https://www.mdpi.com/article/10.3390/gels12030226/s1, Figure S1: SG/CaCl2 composite preparation method; Figure S2: The XRD analysis; Figure S3: ADS modeling flow chart; Figure S4: A comparison of GOR predicted by the developed HDH simulation model at different mass ratios (MR) with the corresponding previous; Table S1: The porosity properties of SG samples [1]; Table S2: D-A isotherm models values; Table S3: Numerical parameters and operation conditions; Table S4: SDWP and COP of the AD system obtained from the present model and previous experimental measurements; Table S5: Validation of the model of the L–V ejector at condenser temperature of 40 °C; Table S6: Deviation between the present results and data given in Ref. [11] for V–V ejector; Table S7: The capital cost of the proposed system; Table S8: Error analysis results to confirm the performance validation of the predictive models of ADS cycle, HDH cycle, LVEJ, and VVEJ; Table S9: Sensitivity analysis of design parameters on adsorption desalination outcomes; Table S10: Sensitivity analysis for hybrid system outcomes. References [54,55,56,57,58,59,60,61] were cited in the Supplementary File.

## Nomenclature

***Symbols***	
A	$Area,\;m^{2}$
C	Adsorption uptake, kg/kg
C_o_	Maximum adsorption uptake, kg/kg
c_p_	Specific heat, J/kg.K
D_s_	$Surface\;diffusivity,\;m^{2}/s$
D_so_	Constant
E	Characteristic energy, J/mol
E_a_	Activation energy, J/mol
ER	Entrainment ratio
F_o_	Constant
h	Specific enthalpy, J/kg
h_fg_	Latent heat of vaporization, J/kg
H_st_	Heat of adsorption, J/mol
i	Interest rate
m	Mass, kg
$\dot{m}$	Mass flow rate, kg/s
n	Isotherm constant
P	Pressure, Pa
$\dot{Q}$	Heat, W
Ṝ	Universal gas constant, J/mol.K
Rp	Particle radius of adsorbent, m
s	Specific entropy, J/kg.K
${\dot{S}}_{gen}$	Entropy generation, W/K
***Greek***	
α	Thermal expansion, 1/K
µ	Chemical potential, J/mol
η	Efficiency, %
ν	kinematic viscosity, m^2^/s
τ	Number of cycles per day, 1/day
***Subscripts***	
1, 2, …	State
Al	Aluminum
ch	Chilled water
co	Condenser
Cu	Copper
cw	Cooling water
des	Desorption
deh	Dehumidifier
dr	Driving
Ej	Ejector
eq	Equilibrium
ev	Evaporator
hex	Hea exchanger
hum	Humidifier
hw	Hot water
ideal	Ideal conditions
in	Inlet
ir	Iron
l	*Labor*
L-V	Liquid–vapor
m/o	Maintenance/operation
out	Outlet
sw	Seawater
T	Total
v	Vapor
V-V	Vapor–vapor
w	Water
***Superscripts***	
AD-co	Adsorbed bed-condenser
AD-ev	Adsorbed bed-evaporator
Ej-co	Ejector-condenser
***Abbreviations***	
AD	Adsorption desalination
EJ	Ejector
F	Capital recovery factor
GOR	Gained output ratio
