# Supplementary material for: Techno-Economic and Environmental Assessment of Solar-Driven Hybrid Adsorption Desalination–HDH Using Silica Gel/Cacl2 Under Saudi Arabian Climate
Source: Gels. 2026 Mar 10;12(3):226. doi: 10.3390/gels12030226 (PMC13025401; doi:10.3390/gels12030226)
Supplement: Supplementary file 1 [file gels-12-00226-s001.zip › gels-4104090-supplementary.pdf]

SUPPLEMENTARY

Techno-economic and environmental assessment of solar-driven hybrid adsorption desalination–HDH using silica gel/$CaCl_2$ under Saudi Arabian climate

Ehab S. Ali[1], Ahmed S. Alsaman[1,*], Ridha Ben Mansour[1], Rached Ben-

[1]Interdisciplinary Research Center for Sustainable Energy Systems, KFUPM, Dhahran, 31261, Saudi Arabia

[2]Mechanical Engineering Department, KFUPM, Dhahran 31261, Saudi Arabia

*Corresponding author: Ahmed S. Alsaman: ahmed.wahballa@kfupm.edu.sa

1. Adsorbent Materials

1.1 Material

Silica gel (SG) was selected as the baseline adsorbent due to its wide availability, non-toxicity, and proven suitability for low-temperature adsorption-driven applications. To enhance surface activity and improve adsorption performance, SG was subjected to acid treatment prior to composite preparation. Acid activation using hydrochloric acid has been widely reported as an effective approach for modifying porous adsorbents and increasing their adsorption capacity and specific surface area [1]. Based on these studies, a concentration of 2 M HCl was adopted as it provides the most favorable improvement in adsorption capacity and BET surface area. Accordingly, 60 cm^3 of 2 M HCl was added to 10 g of SG, and the suspension was stirred at room temperature for 24 h. The treated material was then filtered, thoroughly washed several times to remove residual acid, and dried at 150 °C for 12 h.

The silica gel/$CaCl_2$ composite (SG/$CaCl_2$) was subsequently prepared using a direct impregnation method. In this procedure, $CaCl_2$ was first dissolved in distilled water and then mixed with 2.1 g of dried SG to achieve a salt loading of 25 wt.%. The resulting mixture was stirred at room temperature for 24 h to promote uniform salt distribution within the porous gel structure. After impregnation, the composite was filtered to remove excess solution and then dried at 150 °C for 12 h under atmospheric pressure to eliminate residual moisture and stabilize the composite. The overall workflow of the acid treatment and impregnation steps is illustrated schematically in Fig. 2, while detailed synthesis conditions and characterization protocols are provided in the Supplementary File.

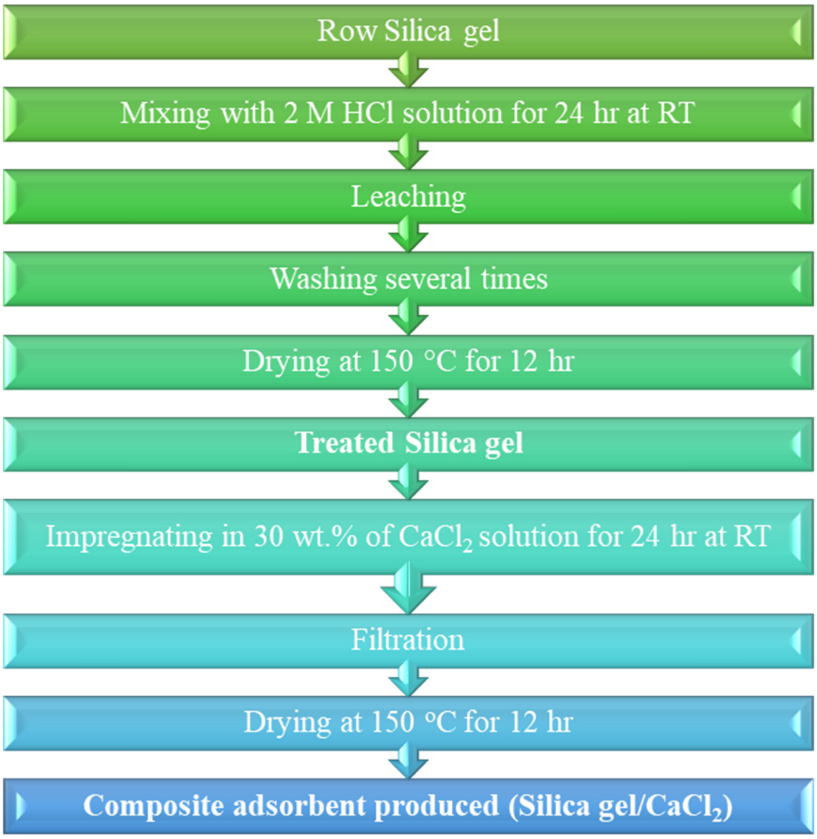

Figure S.1 SG/CaCl2 composite preparation method[1]

1.2 X-ray diffraction

The structural features of raw SG, SG/HCl, and SG/$CaCl_2$ were analyzed using X-ray diffraction (XRD) with a Bruker Axs-D Advance diffractometer employing Cu-Kα radiation (λ = 1.54060 Å) over a 2θ scan range of 20–60°. The experimental procedure follows the methodology reported in Refs. [2,3]. XRD patterns analysis of SG samples is shown in Fig. S.2. From the figure, all samples have wide silicon dioxide (O_2Si) peaks at 22°, indicating the presence of components including SiO_2. The treatment and activation processes have no change in peak curves. Si O_2 peak drop for SG/$CaCl_2$, causing the material to become amorphous.

.

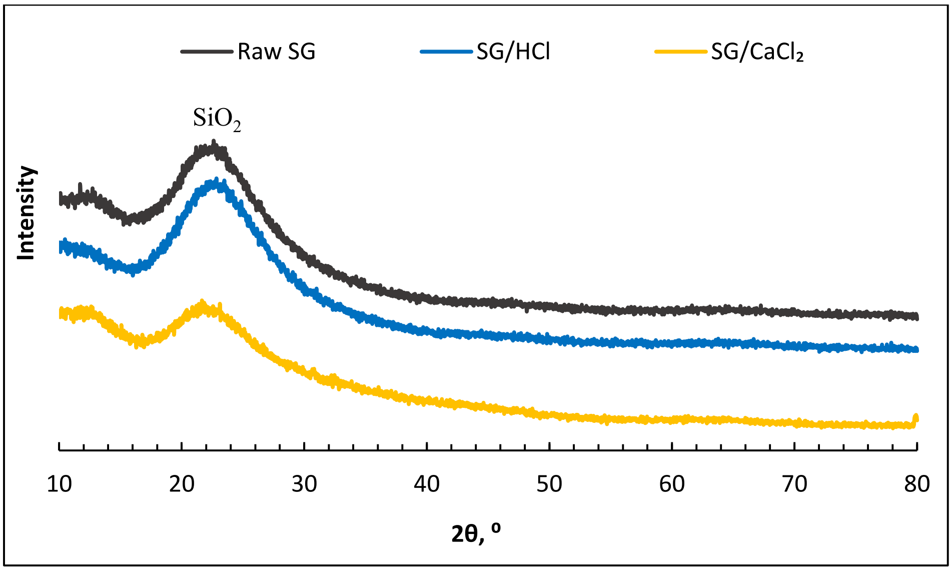

Fig. S.2. The XRD analysis [1].

1.3 Nitrogen adsorption isotherm

Due to the treatment and activation of the raw silica gel (SG), both the specific surface area, S_{BET}, and $V_{0.99}$ increased. In contrast, after incorporating $CaCl_2$ through the composition process, the S_{BET} and $V_{0.99}$ decreased, which can be attributed to partial pore filling by the salt and the resulting obstruction of pore accessibility. **Table S.1** presents the pore size distribution of the raw and modified SG samples, indicating that all materials exhibit pore diameters primarily within the 2–7 nm range. This distribution is consistent with the IUPAC classification, confirming the mesoporous nature of the prepared SG samples[4]. The porosity properties of SG samples are presented in **Table S.1**.

Table S.1: The porosity properties of SG samples[1]:

**Adsorbent**	**S_{BET} (m^2/g)**	**$V_{0.99}$ (cm^3/g)**	**Average pore size (nm)**	**R_p (nm)**
**Raw SG**	266.802	0.554224	4.154	5.111
**SG/HCl**	384.75	0.7311	3.8	3.5442
**SG/$CaCl_2$**	262.43	0.545	4.155	5.1962

1.4 Water vapor adsorption

The extracted D–A parameters and the associated thermodynamic quantities are listed in Table S.2.

Table S.2: D-A isotherm models values [1]

**Adsorbent**	**C_0 (kg/kg)**	**E (kJ/mol)**	**n (-)**	**Average H_{st} (kJ/kg)**
**Raw SG**	0.45	4360	1.69	2890
**SG/HCl**	0.55	4836	1.68	2957
**SG/$CaCl_2$**	0.95	4021	1.39	2782

2. Modelling parameters and Flowcharts

Table S.3: Numerical parameters and operation conditions [5]

**Parameter**	**value**	**Unit**	**Parameter**	**value**	**Unit**
UA_{con}	300	W/K	Cp_{ir}	450	J/kg.K
UA_{bed}	500		Cp_w	4180	
UA_{eva}	300		Cp_v	4190	
M_{bed}	15	kg	R	8314	J/mol.K
M_{con}	7.5		i	5%	-
M_{eva}	7		Y	30	Year
$M_{w,eva}$	3		F	0.9	-
M_{ac}	1.8		W	8.11%	-
M_{SG}	4	kg	β	4.63%	-
Cp_{cu}	386	J/kg.K	$\dot{m}_{ch}$	0.20	kg/s
$\dot{m}_{hw}$	0.20	kg/s	T_h	50-95	°C
$\dot{m}_{cw}$	0.30	kg/s	$T_{chill,in}$	25	°C
T_c	25	°C	t_{cycle}	700	s

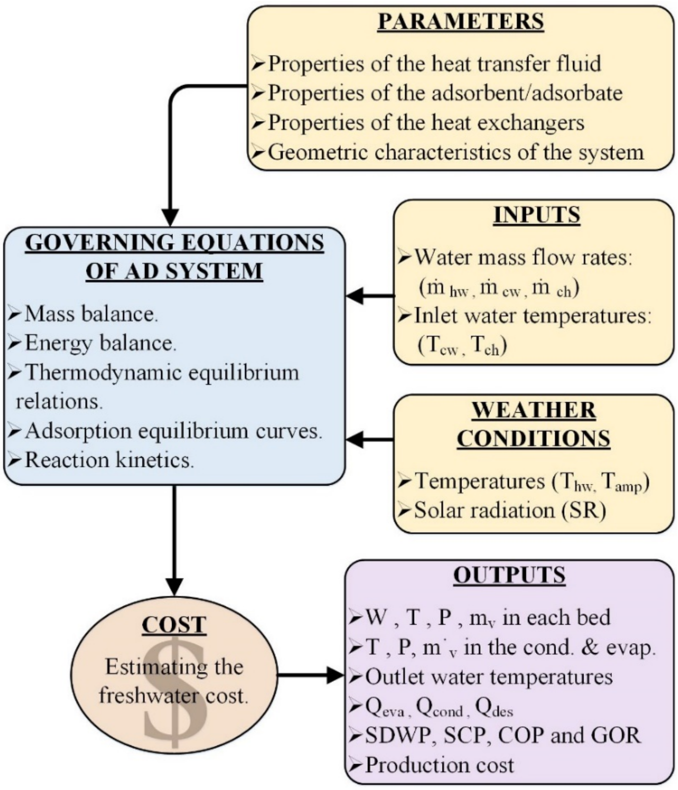

Fig. S.3 ADS modeling flow chart [1].

3. Model Validation

Overall model verification was conducted in three stages by examining (i) the AD model, (ii) the liquid–vapor (L–V) ejector model, and (iii) the vapor–vapor (V–V) ejector model. At each stage, the predicted results were benchmarked against values available in the published literature to ensure consistency with established performance trends. In addition, the entropy generation rate ($\dot{S}_{gen}$) was enforced, ensuring compliance with the first and second laws of thermodynamics and implying positive exergy destruction within each component.

3.1 Validation of the AD model

The present adsorption desalination (AD) numerical model was validated against the experimental measurements reported in Ref under identical operating conditions. Model

predictions were directly compared with the corresponding experimental results from Ref. [6], and the simulated and measured values are summarized in Table S.4. The comparison indicates close agreement between the numerical and experimental outcomes; the maximum deviation is below 5% for SDWP and below 4% for COP. These limited discrepancies demonstrate that the model captures the AD cycle behavior with good accuracy and is therefore suitable for evaluating system performance.

Table S. 4 SDWP and COP of the AD system obtained from the present model and previous experimental measurements [6]

Cycle No.	SDWP (m^3/ton/day)			COP (–)		
	Exp. data	Present data	Error (%)	Exp. data	Present data	Error (%)
1	4.15	4.2	1.20	0.44	0.451	2.50
2	4.01	4.13	2.99	0.448	0.46	2.68
3	3.95	4.07	3.04	0.454	0.472	3.96
4	3.82	4.0	4.71	0.46	0.475	3.26
5	3.8	3.9	2.63	0.453	0.462	1.99
6	3.7	3.8	2.70	0.45	0.457	1.56

3.2 Validation of the liquid to vapor ejector

To validate the liquid-to-vapor (L-V) ejector model, results from the current simulation were compared with those published in Ref. [7] , using R134a as the working fluid. Table S.5 shows the relative error between the present model and the literature values. The average deviation in the outlet pressure of the L-V ejector is found to be less than 0.7%, and the maximum error in the entrainment ratio is just 0.127%. These minimal differences confirm that the model accurately captures the performance of the L-V ejector.

Table S. 5 Validation of the model of the L-V ejector at condenser temperature of 40 °C

T_{ev} (°C)	ER (–)			$P_{EJ\text{-}cond}$ (kPa)		
	Previous data [7]	Present data	Error (%)	Previous data [7]	Present data	Error (%)
10	0.789	0.790	0.127	437.1	435	0.480
5	0.762	0.7625	0.066	375.0	376.25	0.333
0	0.7359	0.7368	0.122	320.4	321.8	0.437

-5	0.712	0.712	0.000	272.4	273.8	0.514
-10	0.6879	0.6873	0.087	230.5	232.0	0.651

3.3 Validation of vapor to vapor ejector

For the vapor-to-vapor (V-V) ejector, the model was validated against the results reported in Ref. [8], with water as the working fluid. Under identical operating conditions, the variation between the model's predictions and the reference data is under 3.0%, as illustrated in Table S. 6. This close agreement confirms the validity of the V-V ejector model and its compatibility with the AD system for evaluating the overall performance of the proposed setup.

Table S. 6 Deviation between the present results and data given in Ref. [8] for V-V ejector

T_{ev} (°C)	T_{co} (°C)	ER (Reported)	ER (present)	Error %
15	80	0.265	0.26	1.887
15	85	0.293	0.29	1.024
15	90	0.318	0.325	2.201
15	95	0.352	0.36	2.273
15	100	0.379	0.39	2.902
15	105	0.41	0.42	2.439
15	110	0.438	0.447	2.055
10	100	0.325	0.318	2.154
10.8	100	0.356	0.347	2.528
11.7	100	0.39	0.395	1.282
12.5	100	0.428	0.434	1.402
13.3	100	0.47	0.475	1.064
14.17	100	0.515	0.502	2.524
15	100	0.564	0.571	1.241

In addition to validating each model, entropy generation in every component was calculated and found to be positive, ensuring that the system complies with both the first and second laws of thermodynamics.

3.4 Validation of the HDH model

Finally, the HDH simulation model developed in this study was validated by benchmarking its predictions against the results reported by Sharqawy et al. [9]. As illustrated in Fig. A.3, the present model reproduces the published trends with very good agreement. The remaining

deviation is minor, with the maximum discrepancy between the current outputs and the reference data not exceeding **2.9%**.

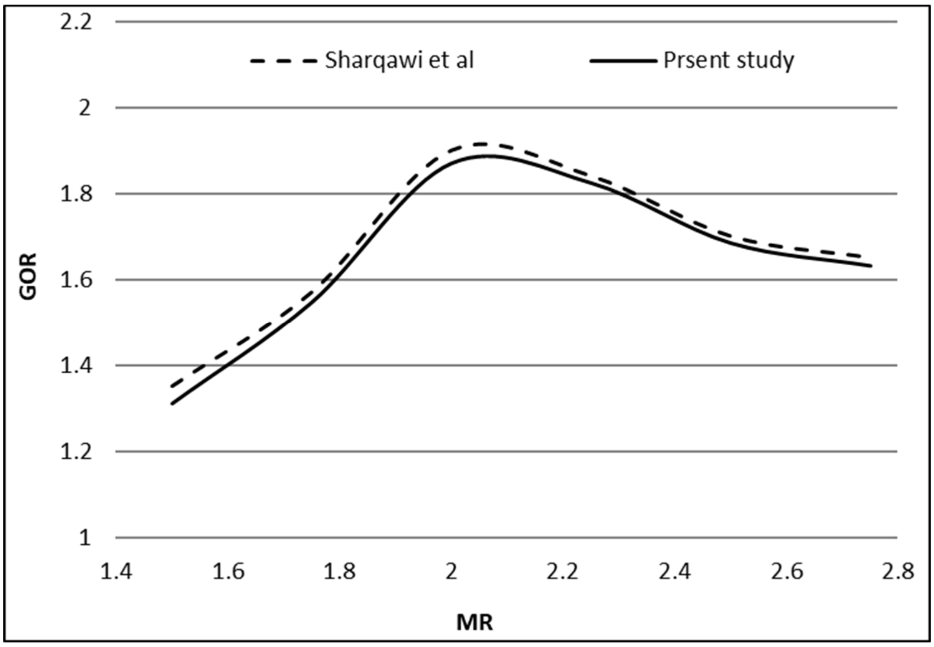

Fig. S.6. A comparison of GOR predicted by the developed HDH simulation model at different mass ratios (MR) with the corresponding previous.

Table S.7. The capital cost of the proposed system.

**AD-EJ-HDH sstem**		
Solar collector	$300 \times No.of\ solar\ collectors$	
Adsorption beds	240	[10]
Adsorbent	68	
Humidifier and dehumidifier	203	
Evaporator	150	
Condenser	120	
Water tanks	250	
Pumps and blowers	345	
Accessories/fittings/pipes	220	
Ejectors	2*200	

4. Error analysis

Error analysis is an essential part of system evaluation because it helps quantify the accuracy and reliability of model predictions. In this study, the validation of the AD–EJ–HDH system sub-models, namely the adsorption desalination (AD) unit, the ejector (EJ) cycle, and the humidification–dehumidification (HDH) cycle, is carried out using two statistical indicators: the root mean square error (RMSE) and the coefficient of determination (R^2). Lower RMSE values reflect smaller prediction deviations and thus greater accuracy, whereas higher R^2 values indicate stronger agreement between predicted and observed results. The corresponding expressions for these metrics are provided below.;

$$R^2 = \frac{\left(\sum_{i=1}^{n_s}(d_i-\bar{d})(y_i-\bar{y})\right)^2}{\sum_{i=1}^{n_s}(d_i-\bar{d})^2 \times \sum_{i=1}^{n_s}(y_i-\bar{y})^2} \quad (1)$$

$$RMSE = \sqrt{\frac{1}{n_s}\sum_{i=1}^{n_s}(d_i - y_i)^2} \quad (2)$$

Table S.8: Error analysis results to confirm the performance validation of the predictive models of ADS cycle, HDH cycle, LVEJ, and VVEJ.

Model	Output parameters	R2	RMSE
**ADS cycle**	Gained output ratio (GOR)	0.9799	0.0120
	Water productivity (L/hr)	0.9654	0.122
**VVEJ**	Entrainment ratio (-)	0.9921	0.0071
**LVEJ**	Entrainment ratio (-)	0.9999	0.0035
**HDH**	GOR	0.9782	0.0756

The developed AD, HDH, and EJ models exhibit strong predictive capability when benchmarked against corresponding experimental data reported in the literature. Across both performance and

thermal indicators, the RMSE values remain consistently low, while the coefficient of determination (R^2) is above 0.965 in most cases, indicating excellent agreement between the simulated outputs and the reference measurements. These results substantiate the robustness of the proposed modeling framework and confirm its reliability for the present analysis.

Table S.8 presents the sensitivity analysis of the adsorption desalination fresh water cost with cost parameters (capital and operations cost). The table shows that the total freshwater cost system is less sensitive to operation costs. The capital cost sensitivity reaches ±9.6% and ±7.9 % when the system is driven by solar energy and waste heat, respectively. The operation cost sensitivity reaches ±2.9 % and ±1.5 % when the system is driven by solar energy and waste heat, respectively.

Table S.9 Sensitivity analysis of design parameters on adsorption desalination outcomes.

Parameters	Freshwater Cost (Powered by waste heat)	Freshwater Cost (Powered by solar energy)
Capital cost ± 10 %	±7.9 %	±9.6%
Electrical pumping cost ± 10 %	±2 %	±0.4 %
Labor cost ± 10 %	±0.6 %	±0.7 %
Maintenance cost ± 10 %	±0.31 %	±0.39 %
Operation cost ± 10 %	±1.5 %	±2.9 %

Table S.10 Sensitivity analysis for hybrid system outcomes.

**Adsorbent**	**Hybrid system SDWP**	**Hybrid system SCP**	**Hybrid system GOR**
cycle time ± 10 %	±1.37 %	±1.38 %	±1.41 %
Ejector ER ± 10 %	**5.5%**	0%	5.5%
HDH desalinate water ± 10%	**1.7%**	0%	**1.7%**

References

[1] A.S. Alsaman, A.A. Askalany, E.M.M. Ibrahim, A.M. Farid, E.S. Ali, M.S. Ahmed, Characterization and cost analysis of a modified silica gel-based adsorption desalination application, J. Clean. Prod. 379 (2022) 134614. https://doi.org/10.1016/j.jclepro.2022.134614.

[2] S. Nasrazadani, S. Hassani, Modern analytical techniques in failure analysis of aerospace, chemical, and oil and gas industries, in: Handbook of Materials Failure

Analysis with Case Studies from the Oil and Gas Industry, Elsevier, 2016: pp. 39–54. https://doi.org/10.1016/B978-0-08-100117-2.00010-8.

[3] W.S. Mohamed, A.M. Abu-Dief, Impact of rare earth europium (RE-Eu3+) ions substitution on microstructural, optical and magnetic properties of CoFe2−xEuxO4 nanosystems, Ceram. Int. 46 (2020) 16196–16209. https://doi.org/10.1016/j.ceramint.2020.03.175.

[4] Z.M. Wang, Nanoporous materials, Handbook of Nanophysics: Functional Nanomaterials 2 (2010) 9-1-9–12. https://doi.org/10.1166/153348802321105860.

[5] M. Ghazy, A.A. Askalany, E.M.M. Ibrahim, A.S.A. Mohamed, E.S. Ali, R. AL-Dadah, Solar powered adsorption desalination system employing CPO-27(Ni), J. Energy Storage 53 (2022) 105174. https://doi.org/10.1016/j.est.2022.105174.

[6] A.S. Alsaman, A.A. Askalany, K. Harby, M.S. Ahmed, Performance evaluation of a solar-driven adsorption desalination-cooling system, Energy 128 (2017) 196–207.

[7] M. Hassanain, E. Elgendy, M. Fatouh, Ejector expansion refrigeration system: Ejector design and performance evaluation, International Journal of Refrigeration (2015). https://doi.org/10.1016/j.ijrefrig.2015.05.018.

[8] B.M. Ziapour, A. Abbasy, First and second laws analysis of the heat pipe/ejector refrigeration cycle, Energy (2010). https://doi.org/10.1016/j.energy.2010.04.016.

[9] M.H. Sharqawy, M.A. Antar, S.M. Zubair, A.M. Elbashir, Optimum thermal design of humidification dehumidification desalination systems, Desalination 349 (2014) 10–21. https://doi.org/10.1016/j.desal.2014.06.016.

[10] E.S. Ali, R.H. Mohammed, N.A.A. Qasem, S.M. Zubair, A. Askalany, Solar-powered ejector-based adsorption desalination system integrated with a humidification-dehumidification system, Energy Convers. Manag. 238 (2021) 114113. https://doi.org/10.1016/j.enconman.2021.114113.
